# Secondary Metabolites of the Endophytic Fungus *Lachnum abnorme* from *Ardisia cornudentata*

**DOI:** 10.3390/ijms17091512

**Published:** 2016-09-08

**Authors:** Hsun-Shuo Chang, Chu-Hung Lin, Yi-Shuan Chen, Hui-Chun Wang, Hing-Yuen Chan, Sung-Yuan Hsieh, Ho-Cheng Wu, Ming-Jen Cheng, Gwo-Fang Yuan, Shan-Yu Lin, Yue-Jin Lin, Ih-Sheng Chen

**Affiliations:** 1 Graduate Institute of Natural Products, College of Pharmacy, Kaohsiung Medical University, Kaohsiung 807, Taiwan; hschang@kmu.edu.tw (H.-S.C.); shelfy25@hotmail.com (Y.-S.C.); wanghc@cc.kmu.edu.tw (Hu.-C.W.); duncanwu762001@hotmail.com (Ho.-C.W.); 2 School of Pharmacy, College of Pharmacy, Kaohsiung Medical University, Kaohsiung 807, Taiwan; chuhung.lin@gmail.com (C.-H.L.); molly0627@hotmail.com (S.-Y.L.); cccwei7569@gmail.com (Y.-J.L.); 3 Bioresource Collection and Research Center, Food Industry Research and Development Institute, Hsinchu 300, Taiwan; chy50@firdi.org.tw (H.-Y.C.); sungyuan@gmail.com (S.-Y.H.); gfy@firdi.org.tw (G.-F.Y.)

**Keywords:** *Lachnum abnorme*, *Ardisia cornudentata*, myrsinaceae, stem, anti-inflammatory activity

## Abstract

Fractionation of an EtOAc-soluble fraction of the solid fermentate of an endophytic fungus, *Lachnum abnorme* Mont. BCRC 09F0006, derived from the endemic plant, *Ardisia cornudentata* Mez. (Myrsinaceae), resulted in the isolation of three new chromones, lachnochromonins D–F (**1**–**3**), one novel compound, lachabnormic acid (**4**), along with nine known compounds (**5**–**13**). Their structures were elucidated by spectroscopic analyses. Alternariol-3,9-dimethyl ether (**6**) was given the correct data as well as 2D spectral analyses for the first time. This is the first report of the isolation of one unprecedented compound **4** from *Lachnum* genus, while all known compounds were also found for the first time from *Lachnum*. The effects of some isolates (**3**, **4**, **7**–**9**, **10**, and **13**) on the inhibition of nitric oxide (NO) production in lipopolysaccharide (LPS)-activated RAW 264.7 murine macrophages were also evaluated. Several compounds exhibited weak inhibitory activity on lipopolysaccharide (LPS)-stimulated NO production in RAW 264.7 macrophages.

## 1. Introduction

**Figure 1:**
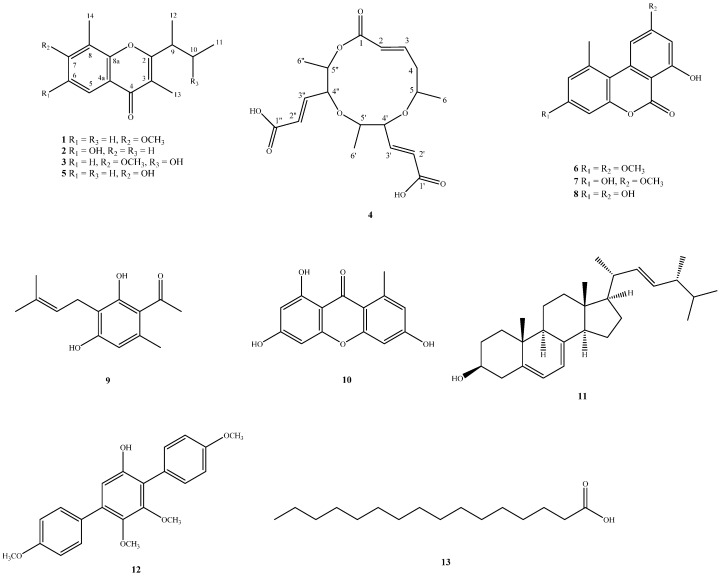
Chemical structures of compounds **1**–**13**.

Plant endophytes are regarded as sorts of particular-biotope microorganisms by residing in the tissues of living plants without causing any immediate obvious negative effects in their host plants [1,2]. Recently, some endophytic fungi have been applied in medicine and agriculture. For example, taxol, an antineoplastic agent, was isolated from *Taxomyces andreanae* derived from *Taxus brevifolia*; crytocandin and cyptocin, antimycotic agents, were separated from *Cryptosporiopsis* cf. *quercina* residing in *Tripterygium wilfordii* [3,4,5]. A series of investigation programs on Formosan endemic plants’ endophytes, some secondary metabolites from fungal strains, showed bioactive potential for exploration. One of these fungal strains, *Lachnum abnorme* Mont. (Hyaloscyphaceae) BCRC 09F0006, was isolated from the stem of an endemic plant, *Ardisia cornudentata* Mez. (Myrsinaceae). In previous chemical investigations of the genus *Lachnum*, three species and one unidentified species were studied, and 35 secondary metabolites were reported, including benzenoids, coumarins, isocoumarins, chromones, phthalides, etc. [6,7,8,9,10,11]. Investigation of the EtOAc-soluble fraction of the solid fermentate from *L*. *abnorme* led to the isolation of four new compounds, lachnochromonins D (**1**), E (**2**), F (**3**), and lachabnormic acid (**4**), along with nine known compounds (**5**–**13**) (Figure 1). The structure identification of the new compounds and the NO inhibitory in macrophage RAW 264.7 cells of the main isolates will be described below.

## 2. Results

### 2.1. Purification and Characterization

The solid state fermentate of *L*. *abnorme* was extracted with methanol and the resulted extracts were partitioned into the EtOAc and H_2_O soluble layers. The EtOAc layer was purified by conventional chromatographic techniques to obtain thirteen compounds (**1**–**13**), and the structures were elucidated by 1D and 2D NMR spectra and compared with literature data.

### 2.2. Structure Elucidation of Compounds ***1***–***4***

**Figure 2:**
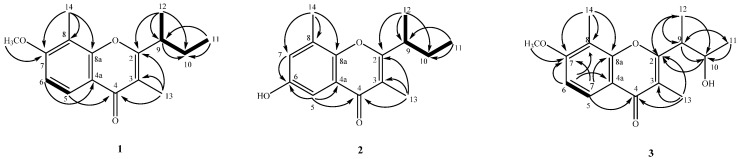
Key HMBC (H→C) and COSY (bold line) correlations for compounds **1**–**3**.

Compound **1** was obtained as an optically active colorless oil with ${\left[\alpha \right]}_{D}^{25}$ −33.6 (*c* 0.47, CHCl_3_), and its molecular formula was established as C_16_H_20_O_3_ by ESI and HRESIMS with seven degrees of unsaturation. The UV absorptions at 231, 246, 254 sh, and 298 nm and IR absorptions at 1632 cm^−1^ (a conjugated carbonyl group), 1604 and 1579 cm^−1^ (an aromatic ring) accompanying the ^13^C NMR resonnance at δ 178.4 (C-4), suggested a chromone skeleton [12]. The ^1^H NMR spectrum showed two aromatic protons with *ortho*-coupling at δ 6.92 (1H, d, *J* = 8.8 Hz, H-6) and 8.03 (1H, d, *J* = 8.8 Hz, H-5), which was assignable due to being next to the carbonyl group of chromone moiety. The fragments of C-12-C-9-C-10-C-11 were observed by COSY analysis. The presence of a *sec*-butyl group was further confirmed by HMBC spectrum showing the correlation between H-12 to C-2, C-9, C-10, and H-11 to C-9, and C-10. The HMBC (Figure 2) spectrum revealed the correlation between H-5 and C-4, C-6 (δ_C_ 108.0), C-7 (δ_C_ 160.6), and C-8a (δ_C_ 156.0); H-6 and C-7, C-8 (δ_C_ 113.3), C-4a (δ_C_ 116.5); OCH_3_ (δ_H_ 3.91) and C-7; H-14 and C-7, C-8, and C-8a; H-9 and C-2, C-3; H-13 and C-2, C-3 and C-4, thus the location of methyl group (δ_H_ 2.27) at C-8, methoxyl group at C-7, another methyl group (δ_H_ 2.05) at C-3 and the connection of a *sec*-butyl group to C-2 can be confirmed. The ^1^H NMR spectrum (Table 1) of **1** was similar to that of lachnochromonin C (**5**) [13], also obtained in this study, except a methoxy group at C-7 in (**1**) was in place of a hydroxy group (δ_H_ 8.91, br s) in **5**. According to the above data, the structure of **1** was unequivocally determined as 2-*sec*-butyl-7-methoxy-3,8-dimethyl-4*H*-chromen-4-one, which was further confirmed by COSY (Figure 2), ^13^C NMR (Table 1), DEPT, HSQC, HMBC (Figure 2), and NOESY techniques, and designated as lachnochromonin D. Hence, compound **1** had the proposed structure, leaving the C-9 configuration undetermined.

Compound **2** was obtained as colorless needles with ${\left[\alpha \right]}_{D}^{25}$ −57.2 (*c* 0.17, CHCl_3_). The ESIMS and HRESIMS were used to establish the molecular formula of **2** as C_15_H_18_O_3_ with seven degrees of unsaturation. The UV absorptions at 224, 245, 251, and 299 nm and after addition of KOH aqueous. solution gave a bathochromic shift. The IR absorption at 3194 cm^−1^ (a hydroxy group), 1626 cm^−1^ (a conjugated carbonyl group), 1584 and 1482 cm^−1^ (an aromatic ring) suggested the presence of a phenolic chromone skeleton [12], which was also supported by a ^13^C NMR resonance of C-4 (δ_C_ 178.3) and δ_H_ 7.35 (1H, br s, OH-6) in ^1^H NMR spectrum. The ^1^H NMR data was similar to that of **5** except two *meta*-coupled protons of H-5 (δ 6.90) and H-7 (δ 7.96), one hydroxy group in C-6 of **2** replaced two *ortho*-coupled protons of H-5 and H-6, and one hydroxy group (δ 8.91, br s) in **5**. Thus, the structure of **2** was proposed to be 2-*sec*-butyl-6-hydroxy-3,8-dimethyl-4*H*-chromen-4-one, which was further confirmed by COSY (Figure 2), ^13^C NMR (Table 1), DEPT, HSQC, HMBC (Figure 2), and NOESY experiments, and namely lachnochromonin E. At present, the absolute configuration at C-9 of **2** remains uncertain.

Compound **3** was obtained as an optically active yellowish powder with ${\left[\alpha \right]}_{D}^{25}$ –68.5 (*c* 0.22, CHCl_3_). The molecular formula was established as C_16_H_20_O_4_ by the ESIMS and HRESIMS with seven degrees of unsaturation. Its UV spectra showed absorption bands at 228, 244, 252, and 297 nm, and the IR spectrum revealed the presence of a hydroxy group (3397 cm^−1^), and a conjugated carbonyl group (1627 cm^−1^). Analyses of the ^1^H NMR spectrum indicated the presence of two aromatic protons with *ortho*-coupling at δ 6.94 (1H, d, *J* = 8.9 Hz, H-6) and 8.03 (1H, d, *J* = 8.9 Hz, H-5), one methoxy group at δ 3.94 (3H, s, OCH_3_-7), four methyl groups at δ 1.32 (3H, d, *J* = 7.2 Hz, H-12), 1.34 (3H, d, *J* = 6.0 Hz, H-11), 2.09 (3H, s, H-13), and 2.29 (3H, s, H-14), one methine and one oxymethine at δ 3.11 (1H, qd, *J* = 7.2, 6.6 Hz, H-9) and 4.15 (1H, dq, *J* = 6.6, 6.0 Hz, H-10), respectively. Meanwhile, the ^13^C NMR spectrum exhibited 16 signals that were clearly classified into four methines, five methyls, and seven quaternary carbons based on the HSQC, suggesting **3** might be a chromone, similar to **1**, which was corroborated by its 2D NMR spectra involving COSY, HSQC, HMBC and NOESY. The only difference was that one CHOH group (δ 4.15 (1H, dq, *J* = 6.6, 6.0 Hz, H-10)) attached on C-10 in **3**, replacing a CH_2_ group (δ 1.68/1.82 (each 1H, m, CH_2_-10)) at C-10 in **1**, which was further confirmed in this proposed structure by the 2D NMR data (COSY and HMBC) (Figure 2), allowed determining the structure of **3**. The configurations of C-9 and C-10 in **3** remain uncertain. Thus, the structure of **3** was fully established and named lachnochromonin F.

**Figure 3:**
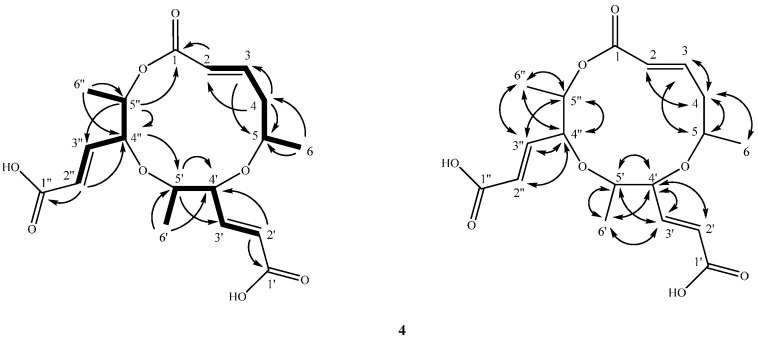
Key HMBC (H→C), COSY (bold line), and NOESY (H↔H) correlations for compound **4**.

Compound **4** was isolated as a whitish powder with ${\left[\alpha \right]}_{D}^{25}$ ±0° (*c* 0.38, MeOH). The ESIMS and HRESIMS data provided the molecular formula C_18_H_24_O_8_ with seven degrees of unsaturation. The UV spectrum exhibited the absorption at 260 nm. The IR spectrum showed absorptions at 3455, 1720 cm^−1^ for the OH group and the carbonyl group, respectively. The ^1^H NMR, ^13^C NMR, DEPT, and HSQC spectra displayed signals for three methyl doublets at δ 1.28 (3H, d, *J* = 6.0 Hz, H-6)/21.2 (C-6), 1.31 (3H, d, *J* = 6.0 Hz, H-6′)/18.4 (C-6′), and 1.34 (3H, d, *J* = 6.0 Hz, H-6′′)/18.5 (C-6′′), three *trans*-form olefinic groups at δ 5.83 (1H, ddd, *J* = 15.6, 1.8, 0.9 Hz, H-2)/125.3 (C-2), 6.86 (1H, ddd, *J* = 15.6, 9.6, 5.9 Hz, H-3)/146.3 (C-3), 5.93 (1H, dd, *J* = 15.9, 0.9 Hz, H-2′)/123.95 (C-2′), 6.81 (1H, dd, *J* = 15.9, 7.6 Hz, H-3′)/149.1 (C-3′), 5.97 (1H, dd, *J* = 15.7, 1.2 Hz, H-2′′)/123.98 (C-2′′), and 6.83 (1H, dd, *J* = 15.7, 7.2 Hz, H-3′′)/148.5 (C-3′′), one non-equivalent CH_2_ at δ 2.43 (1H, dddd, *J* = 14.4, 9.6, 9.6, 0.9 Hz, H-4b), 2.59 (1H, dddd, *J* = 14.4, 5.9, 3.0, 1.8 Hz, H-4a)/40.0 (C-4), five oxymethines at δ 5.10 (1H, dqd, *J* = 9.6, 6.0, 3.0 Hz, H-5)/70.3 (C-5), 4.12 (1H, m, H-4′)/75.2 (C-4′), 4.84 (1H, m, H-5′)/73.1 (C-5′), 4.17 (1H, m, H-4′′)/74.7 (C-4′′), 4.82 (1H, m, H-5′′)/73.9 (C-5′′), and three carbonyl groups at δ 165.8 (C-1), 165.9 (C-1′), and 166.1 (C-1′′). The above observation, followed by the COSY (Figure 3) and HSQC spectrum of **4,** established the presence of the partial three substitutes: (**A**) C-1 to C-6, (**B**) C-1′ to C-6′, and (**C**) C-1′′ to C-6′′, for skeleton of **4**. The entire skeleton of **4** was constructed by the aid of the HMBC spectrum (Figure 3). In the HMBC spectrum of **4** (Figure 3), there were ^2^*J*, ^3^*J*-correlations from H-2 to C-1, C-3, and C-4; from H-3 to C-1, C-2, C-4, and C-5; from H-4 to C-2, C-3, and C-5; from H-6 to C-4 and C-5; from H-2′ to C-1′ and C-4′; from H-3′ to C-4′ and C-5′; from H-4′ to C-2′, C-3′, C-5′, and C-6′; from H-6′ to C-4′ and C-5′; from H-2′′ to C-1′′, C-3′′, and C-4′′; from H-3′′ to C-4′′ and C-5′′; from H-4′′ to C-2′′, C-3′′, C-5′′, and C-6′′, H-6’ to C-4’ and C-5’. Moreover, the key HMBC correlations of H-4′′/ C-5′; H-5′′/C-1; H-4′/C-5 verified the junction of the each two substitutes **A** to **B** unit at C-5, **B** to **C** unit at C-5′, and **C** to **A** at C-5′′ through the oxygen atom between C-5/C-4′, C-5′/C-4′′, and C-5′′/C-1, respectively. This structure was composed of three (*E*)-5-hydroxy-2-hexenoic acid groups. Unfortunately, the NOESY (Figure 3) spectrum only showed the correlations within the fragments, and therefore the relative configurations of **4** could not be determined. Because of the optical inactivity, **4** was also proposed to be racemic. ^1^H and ^13^C NMR, COSY (Figure 3), HMBC (Figure 3), and NOESY (Figure 3) experiments confirmed the planar structure as **4** and was named as a lachabnormic acid.

Alternariol-3,9-dimethyl ether (**6**) was a known compound that was isolated in this study. The ^1^H NMR data of compound **6** was compared with the two reference data [14,15] very closely, but only the chemical shift of H-11 in compound **6** is different. In Yang’s report [15], δ 2.03 (Me-11) was too high field to be assigned as a methyl group in ring A of 6*H*-benzo[*c*]chromen-6-one skeleton in comparison with the reference compounds alternariol-9-methyl ether (**7**) [16,17] and alternariol (**8**) [18]. The chemical shift of H-11 of compound **6** should appear at δ 2.81, and the chemical shift of H-11 in the reference [15] displayed at δ 2.03 is erroneous. We had asked the corresponding author [15] to provide us with their original ^1^H and ^13^C NMR spectrum of compound **6** for further confirmation. It was caused by the author’s typo. According to the donated spectrum, the chemical shift of H-11 of **6** indeed appeared at δ 2.81 (in CDCl_3_). We also provided the correct data and 2D NMR spectrum to further confirm the structure.

The known compounds, lachnochromonin C (**5**) [13], alternariol-9-methyl ether (**7**) [16,17], alternariol (**8**) [18], pestalorionol (**9**) [19], 1,3,6-trihydroxy-8-methylxanthone (**10**) [20], ergosterol (**11**) [21], 6′-hydroxy-4,2′,3′,4′′-tetramethoxy-*p*-terphenyl (**12**) [22], and palmitic acid (**13**) [23] were identified by comparison of their physical and spectroscopic data with values reported in the literature.

A literature review revealed that lachnochromonin C (**5**) has been reported in the patent literature concerning its strong anti-inflammatory activity [13]. In our study, we tested the anti-inflammatory activity by measuring the nitric oxide (NO) production in lipopolysaccharide (LPS)-activated RAW 264.7 macrophages. The iNOS inhibitory activity of compounds **3**, **4**, **7**, **8**, **9**, **10**, and **13** at a concentration of 100 μM was presented in Table 2, showing most compounds had nitrite production values of 77.75%–101.83% under LPS induction. The result indicated that all compounds that we tested exhibited a weak anti-inflammatory activity. The increase in cell viability of compound **8** suggests that this compound might have some activities in cell proliferation, which is worthy to be explored by other evaluation models.

## 3. Discussion

Natural products from medicinal plants and microorganisms are the most concordant and productive source for the discovery of new pharmaceuticals and lead compounds. Recently, a great deal of interest has been generated by discovery of remarkable pharmacological agents from endophytic fungi. In our ongoing pharmacological screening program on biodiversity of endophytic fungi from endemic Formosana plants, we found out that the extracts of an endophytic fungus of the ascomycetes family growing on an endemic plant, *A. cornudentata* Mez. (Myrsinaceae), named *L. abnorme*, showed approximately 95% inhibition at a concentration of 10 μg/mL. It is remarkable to inhibit lipopolysaccharide (LPS)-induced nitric oxide (NO) release in RAW 264.7 murine macrophages. Careful investigation the EtOAc-soluble fraction of the solid fermentate from *L*. *abnorme* led to the isolation of three new chromones, lachnochromonins D–F (**1**–**3**), and one novel compound, lachabnormic acid (**4**), together with nine known isolates. Alternariol-3,9-dimethyl ether (**6**) was given the correct data as well as 2D spectral analyses for the first time. Interestingly, the unique side chain 3-hydroxybutan-2-yl in lachnochromonin F (**3**) so far was only isolated from this plant. Various substitutents of the bulky or small side chain [24] at the C-2 position of chromone isolated from different species indicated the distinct and diverse metabolic pathway of *Lachnum*. To our knowledge, the chromone skeleton has seldom been previously reported in the genus *Lachnum*. This type of compound could stimulate future phytochemical studies.

## 4. Materials and Methods

### 4.1. General

General. TLC: silica gel 60 F_254_ precoated plates (Merck, Kenilworth, NJ, USA). Column chromatography (CC): silica gel 60 (70–230 or 230–400 mesh, Merck) and Spherical C18 100A Reversed Phase Silica Gel (RP-18) (particle size: 20–40 μm) (Silicycle). UV Spectra: Jasco V-530 UV/VIS spectrophotometer (Tokyo, Japan); λ_max_ (log ε) in nm. Optical rotation: Jasco P-2000 polarimeter; in CHCl_3_. IR Spectra: Jasco FTIR-4200 spectrophotometer; ν in cm^−1^. ^1^H, ^13^C and 2D NMR spectra: Varian-VNMRS-600 (Palo Alto, CA, USA), and Varian-Mercury-400 spectrometers; δ in ppm rel. to Me_4_Si, *J* in Hz. EI–MS: VG-Biotech Quatro-5022 mass spectrometer (Palo Alto, CA, USA); *m*/*z* (rel. %). ESI and HRESIMS: Bruker APEX-II mass spectrometer (Billerica, MA, USA); in *m*/*z*.

### 4.2. Fungus Material

The fungal strain *Lachnum abnorme* was isolated from the stem of *Ardisia cornudentata*, which was collected in Wutai Township, Pingtung County, Taiwan, during August of 2008, and identified by Ih-Sheng Chen, one of the authors. The fungal strain was identified as *L.* *abnorme* (family Hyaloscyphaceae) by Sung-Yuan Hsieh, one of the authors, basing on cultural and anamorphic data.

Fungus cultivation and fermentation were carried out by a previously reported method [25], and the sequence data derived from the fungal strain has been submitted and deposited at GenBank with accession no. AB267638. BLAST (nucleotide sequence comparison program) search result displayed that the sequence was the most resembling (100%) to the sequence of *L. abnorme*. The strain is preserved at the Bioresource Collection and Research Center (BCRC) of the Food Industry Research and Development Institute (FIRDI), under the ID No. 09F0006.

### 4.3. Extraction and Isolation

Solid fermentate of *L. abnorme* (5 kg) was extracted with cold MeOH to yield a MeOH extract (150 g) which was partitioned in EtOAc–H_2_O (1:1) to produce an EtOAc layer (20 g) and an H_2_O layer. The H_2_O layer was further partitioned with BuOH, and the BuOH layer (50 g) was separated. The EtOAc layer was subjected to C.C. (silica gel; hexane/acetone gradient) to get eight fractions (Frs. 1–8). Fr. 3 was subjected to MPLC (silica gel; hexane/acetone, 5:1) to produce 18 fractions, Frs. 3.1–3.18. Fr. 3.7 was subjected to MPLC (silica gel; hexane/CH_2_Cl_2_/acetone, 30:1:1) to produce 10 fractions, Frs. 3.7.1–3.7.10. Fr. 3.7.7 furnished **13** (170 mg). Fr. 4 was subjected to MPLC (silica gel; CH_2_Cl_2_/acetone, 30:1) to produce 11 fractions, Frs. 4.1–4.11. Fr. 4.3 was subjected to MPLC (silica gel; CH_2_Cl_2_/EtOAc, 100:1) to produce six fractions, Frs. 4.3.1–4.3.6. Fr. 4.3.3 was subjected to MPLC (silica gel; hexane/EtOAc, 10:1) to produce 12 fractions, Frs. 4.3.3.1–4.3.3.12. Fr. 4.3.3.7 was further purified with prep. TLC (hexane/acetone, 15:1) to obtain **9** (*R*_f_ 0.45; 2.2 mg). Fr. 4.3.3.10 was further purified with prep. TLC (hexane/acetone, 10:1) to obtain **6** (*R*_f_ 0.48; 0.9 mg). Fr. 4.4 was subjected to MPLC (silica gel; hexane/EtOAc, 4:1) to produce 11 fractions, Frs. 4.4.1–4.4.11. Fr. 4.4.2 was furnished **1** (45 mg). Fr. 5 was recrystallized from acetone and CH_3_OH to gain **11** (140 mg). Fr. 6 was subjected to MPLC (silica gel; hexane/acetone, 4:1) to produce 10 fractions, Frs. 6.1–6.10. Fr. 6.5 was subjected to MPLC (RP–18; MeOH/H_2_O 1:1) to produce six fractions, Fr. 6.5.1–6.5.6. Fr. 6.5.4 was further purified to MPLC (silica gel; CH_2_Cl_2_/MeOH, 50:1) to yield **2** (4.8 mg). Fr. 6.5.6 was purified to MPLC (silica gel; CH_2_Cl_2_/MeOH, 50:1) to afford **12** (3.3 mg). Fr. 7 was subjected to MPLC (silica gel; hexane/EtOAc, 1:1) to produce 18 fractions, Frs. 7.1–7.18. Fr. 7.5 was subjected to MPLC (silica gel; hexane/acetone, 2:1) to produce 11 fractions, Frs. 7.5.1–7.5.11. Fr. 7.5.2 was subjected to MPLC (silica gel; CHCl_3_/acetone, 30:1) to produce 22 fractions, Frs. 7.5.2.1–7.5.2.22. Fr. 7.5.2.7 afforded **7** (6.2 mg). Fr. 7.5.2.15 was subjected to MPLC (silica gel; CHCl_3_/acetone, 40:1) to produce six fractions, Frs. 7.5.2.15.1–7.5.2.15.6. Fr. 7.5.2.15.4 afforded **5** (152 mg). Fr. 7.5.5 was subjected to MPLC (silica gel; hexane/acetone, 4:1) to produce five fractions, Frs. 7.5.5.1–7.5.5.5. Fr. 7.5.5.2 was subjected to MPLC (silica gel; CHCl_3_/CH_3_OH, 60:1) to produce five fractions, Frs. 7.5.5.2.1–7.5.5.2.5. Fr. 7.5.5.2.5 was subjected to MPLC (silica gel; hexane/acetone, 5:2) to produce four fractions, Frs. 7.5.5.2.5.1–7.5.5.2.5.4. Fr. 7.5.5.2.5.2 was subjected to MPLC (silica gel; CH_2_Cl_2_/CH_3_OH, 40:1) to produce seven fractions, Frs. 7.5.5.2.5.2.1–7.5.5.2.5.2.7. Fr. 7.5.5.2.5.2.6 yielded **10** (3.7 mg). Fr. 7.6 was purified with TLC (RP–18; acetone/H_2_O 2:1) to obtain **8** (*R*_f_ 0.58; 2.1 mg) Fr. 7.9 was subjected to MPLC (silica gel; CHCl_3_/CH_3_OH, 40:1) to produce 22 fractions, Frs. 7.9.1–7.9.22. Fr. 7.9.7 was purified with TLC (RP–18; acetone/H_2_O 1:1) to obtain **3** (*R*_f_ 0.38; 2.2 mg) Fr. 7.9.14 was subjected to MPLC (silica gel; CH_2_Cl_2_/CH_3_OH, 30:1) to produce 10 fractions, Frs. 7.9.14.1–7.9.14.10. Fr. 7.9.14.4 was purified with TLC (RP–18; acetone/H_2_O 2:1) to obtain **4** (*R*_f_ 0.68; 3.8 mg).

#### 4.3.1. Lachnochromonin D (**1**)

Colorless oil; ${\left[\alpha \right]}_{D}^{25}$ −33.6 (*c* 0.47, CHCl_3_); UV λ_max_ (MeOH) (log ε) 231 (4.34), 246 (4.11), 254 sh (4.06), 298 (4.09) nm; IR ν_max_ (ATR) 1632 (C=O), 1604, 1579 (aromatic ring) cm^−1^; ^1^H and ^13^C NMR: see Table 1; ESIMS: *m*/*z* 261 ([M + H]^+^); HRESIMS: *m*/*z* 261.14841 ([M + H]^+^, calcd for C_16_H_21_O_3_, 261.14852).

#### 4.3.2. Lachnochromonin E (**2**)

Colorless needles (MeOH-CH_2_Cl_2_); M.p.: 231–233 °C; ${\left[\alpha \right]}_{D}^{25}$ −57.2 (*c* 0.17, CHCl_3_); UV *λ*_max_ (MeOH) (log ε) 224 (4.30), 245 (4.05), 251 (4.05), 299 (4.07) nm; UV λ_max_ (MeOH + KOH) (log ε) 215 (4.39), 254 (4.25), 338 (4.13) nm; IR ν_max_ (ATR) 3194 (OH), 1626 (C=O), 1584, 1482 (aromatic ring) cm^−1^; ^1^H and ^13^C NMR: see Table 1; ESIMS: *m*/*z* 247 ([M + H]^+^); HRESIMS: *m*/*z* 247.13266 ([M + H]^+^, calcd for C_15_H_19_O_3_, 247.13287).

#### 4.3.3. Lachnochromonin F (**3**)

Yellowish powder; ${\left[\alpha \right]}_{D}^{25}$ −68.5° (*c* 0.22, CHCl_3_); UV λ_max_ (MeOH) (log *ε*): 228 (3.90), 244 (3.62), 252 sh (3.57), 297 (3.61) nm; IR ν_max_ (KBr): 3397 (OH), 1627 (C=O) cm^−1^; ^1^H and ^13^C NMR: see Table 1; ESIMS: *m*/*z* 277 [M + H]^+^; HRESIMS: *m*/*z* 277.1432 ([M + H]^+^, calcd for C_16_H_21_O_4_, 277.1434).

#### 4.3.4. Lachabnormic Acid (**4**)

Whitish powder; ${\left[\alpha \right]}_{D}^{25}$ ±0° (*c* 0.38, MeOH); UV λ_max_ (MeOH) (log ε): 260 (2.88) nm; IR ν_max_ (KBr): 3455 (OH), 1720 (C=O) cm^−1^; ^1^H NMR (CDCl_3_, 600 MHz) δ 1.28 (3H, d, *J* = 6.0 Hz, H-6), 1.31 (3H, d, *J* = 6.0 Hz, H-6′), 1.34 (3H, d, *J* = 6.0 Hz, H-6′′), 2.43 (1H, dddd, *J* = 14.4, 9.6, 9.6, 0.9 Hz, H-4b), 2.59 (1H, dddd, *J* = 14.4, 5.9, 3.0, 1.8 Hz, H-4a), 4.12 (1H, m, H-4′), 4.17 (1H, m, H-4′′), 4.82 (1H, m, H-5′′), 4.84 (1H, m, H-5′), 5.10 (1H, dqd, *J* = 9.6, 6.0, 3.0 Hz, H-5), 5.83 (1H, ddd, *J* = 15.6, 1.8, 0.9 Hz, H-2), 5.93 (1H, dd, *J* = 15.9, 0.9 Hz, H-2′), 5.97 (1H, dd, *J* = 15.7, 1.2 Hz, H-2′′), 6.81 (1H, dd, *J* = 15.9, 7.6 Hz, H-3′), 6.83 (1H, dd, *J* = 15.7, 7.2 Hz, H-3′′), 6.86 (1H, ddd, *J* = 15.6, 9.6, 5.9 Hz, H-3); ^13^C NMR (CDCl_3_, 150 MHz) δ: 18.4 (C-6′), 18.5 (C-6′′), 21.2 (C-6), 40.0 (C-4), 70.3 (C-5), 73.1 (C-5′), 73.9 (C-5′′), 74.7 (C-4′′), 75.2 (C-4′), 123.95 (C-2′), 123.98 (C-2′’), 125.3 (C-2), 146.3 (C-3), 148.5 (C-3′′), 149.1 (C-3′), 165.8 (C-1), 165.9 (C-1′), 166.1 (C-1′′); ESIMS: *m/z* 369 [M + H]^+^; HRESIMS: *m*/*z* 391.1367 ([M + Na]^+^ , calcd for C_18_H_24_O_8_Na, 391.1369).

#### 4.3.5. Lachnochromonin C (**5**)

Colorless needles; M.p.: 160–162 °C; ${\left[\alpha \right]}_{D}^{25}$ +5.66° (*c* 0.06 MeOH); UV λ_max_ (MeOH) (log ε): 229 (4.61), 246 (4.49), 253 sh (4.45), 298 (4.39) nm; UV λ_max_ (MeOH + KOH) (log ε): 221 sh (4.55), 260 (4.62), 337 (4.30) nm; ^1^H NMR (CDCl_3_, 400 MHz) δ 0.93 (3H, dd, *J* = 7.6, 7.2 Hz, H-11), 1.33 (3H, d, *J* = 6.8 Hz, H-12), 1.68 (1H, m, H-10b), 1.85 (1H, m, H-10a), 2.11 (3H, s, H-13), 2.35 (3H, s, H-14), 3.05 (1H, m, H-9), 7.03 (1H, d, *J* = 8.8 Hz, H-6), 7.90 (1H, d, *J* = 8.8 Hz, H-5), 8.91 (1H, br s, OH-7, D_2_O exchangeable); ^13^C NMR (CDCl_3_, 100 MHz) δ: 7.9 (C-14), 9.6 (C-13), 12.0 (C-11), 18.0 (C-12), 27.8 (C-10), 37.8 (C-9), 114.2 (C-6), 111.4 (C-8), 115.1 (C-3), 115.6 (C-4a), 123.8 (C-5), 155.9 (C-8a), 159.5 (C-7), 168.2 (C-2), 179.2 (C-4); ESIMS: *m*/*z* 247 [M + H]^+^.

#### 4.3.6. Alternariol-3,9-dimethyl Ether (**6**)

Colorless needles; M.p.: 179–181 °C; UV λ_max_ (MeOH) (log ε): 254 (4.35), 286 (3.63), 297 (3.61), 331 (3.66) nm; UV λ_max_ (MeOH + KOH) (log ε): 250 (417), 304 (3.46), 354 (3.60) nm; IR ν_max_ (KBr): 3400 (OH), 1669 (C=O) cm^−^^1^; ^1^H NMR (CDCl_3_, 600 MHz) δ 2.81 (3H, s, H-11), 3.86 (3H, s, OCH_3_-3), 3.92 (3H, s, OCH_3_-9), 6.55 (1H, d, *J* = 1.5 Hz, H-8), 6.75 (1H, d, *J* = 2.4 Hz, H-2), 6.76 (1H, d, *J* = 2.4 Hz, H-4), 7.27 (1H, d, *J* = 1.5 Hz, H-10), 12.0 (1H, s, OH-7); ^13^C NMR (CDCl_3_, 150 MHz) δ 25.7 (C-11), 55.5 (OCH_3_-3), 55.7 (OCH_3_-9), 98.9 (C-8), 99.4 (C-6a), 100.0 (C-4), 104.5 (C-10), 110.9 (C-10b), 116.9 (C-2), 138.0 (C-10a), 138.2 (C-1), 153.2 (C-4a), 160.0 (C-3), 165.2 (C-6), 165.4 (C-7), 166.4 (C-9); ESIMS *m*/*z* 287 [M + H]^+^.

#### 4.3.7. Alternariol-9-methyl Ether (**7**)

Colorless needles; M.p.: 245–248 °C; UV λ_max_ (MeOH) (log ε): 219 sh (3.74), 256 (3.99), 288 (3.37), 299 (3.35), 340 (3.44) nm; UV λ_max_ (MeOH + KOH) (log ε): 265 (3.84), 277 sh (3.73), 311 (3.41), 365 (3.41) nm; IR ν_max_ (ATR): 3314 (OH), 1711 (C=O) cm^−^^1^; ^1^H NMR (Acetone-*d*_6_, 400 MHz) δ 2.80 (3H, s, H-11), 3.97 (3H, s, OCH_3_-9), 6.57 (1H, d, *J* = 2.2 Hz, H-8), 6.70 (1H, d, *J* = 2.8 Hz, H-4), 6.80 (1H, d, *J* = 2.8 Hz, H-2), 7.30 (1H, d, *J* = 2.2 Hz, H-10), 9.22 (1H, br s, OH-3), 11.8 (1H, s, OH-7); ^13^C NMR (Acetone-*d*_6_, 100 MHz) δ 26.3 (C-11), 56.9 (OCH_3_-9), 100.5 (C-6a), 100.6 (C-8), 103.4 (C-4), 105.2 (C-10), 111.3 (C-10b), 119.1 (C-2), 139.8 (C-10a), 140.3 (C-1), 154.8 (C-4a), 160.0 (C-3), 166.7 (C-6, C-7), 168.2 (C-9); ESIMS *m*/*z* 273 [M + H]^+^.

#### 4.3.8. Alternariol (**8**)

Yellowish powder; UV λ_max_ (MeOH) (log ε): 254 (3.93), 286 (3.45), 331 sh (3.34) nm; UV λ_max_ (MeOH + KOH) (log ε): 276 (3.92), 317 sh (3.49), 346 sh (3.44) nm; IR ν_max_ (ATR): 3350 (OH), 1657 (C=O) cm^−^^1^; ^1^H NMR (CDCl_3_, 600 MHz) δ 2.77 (3H, s, H-11), 6.37 (1H, d, *J* = 1.8 Hz, H-8), 6.62 (1H, d, *J* = 2.5 Hz, H-4), 6.70 (1H, d, *J* = 2.5 Hz, H-2), 7.27 (1H, d, *J* = 1.8 Hz, H-10); ^13^C NMR (CDCl_3_, 150 MHz) δ 25.8 (C-11), 99.1 (C-6a), 102.0 (C-8), 102.8 (C-4), 105.5 (C-10), 111.0 (C-10b), 118.6 (C-2), 139.8 (C-1), 140.1 (C-10a), 154.5 (C-4a), 159.9 (C-3), 166.2 (C-7), 166.9 (C-9), 167.0 (C-6); ESIMS *m*/*z* 259 [M + H]^+^.

#### 4.3.9. Pestalotionol (**9**)

Yellowish oil; UV λ_max_ (MeOH) (log ε): 219 sh (3.81), 295 (3.61) nm; UV λ_max_ (MeOH + KOH) (log ε): 252 (3.51), 338 (3.90) nm; IR ν_max_ (ATR): 3401 (OH), 1648 (C=O) cm^−^^1^; ^1^H NMR (CDCl_3_, 600 MHz) δ 1.75 (3H, br s, H-10), 1.81 (3H, br s, H-11), 2.46 (3H, s, H-13), 2.64 (3H, s, H-14), 3.41 (2H, d, *J* = 7.5 Hz, H-7), 5.26 (1H, br t, *J* = 7.5 Hz, H-8), 5.83 (1H, br s, OH-4, D_2_O exchangeable), 6.23 (1H, s, H-5), 12.17 (1H, s, OH-2, D_2_O exchangeable); ^13^C NMR (CDCl_3_, 150 MHz) δ 13.1 (C-13), 17.9 (C-11), 22.1 (C-7), 24.6 (C-14), 25.8 (C-10), 111.70 (C-5), 111.74 (C-3), 115.4 (C-1), 121.4 (C-8), 135.4 (C-9), 138.8 (C-6), 158.9 (C-4), 160.5 (C-2), 198.2 (C-12); ESIMS *m*/*z* 273 [M + K]^+^.

#### 4.3.10. 1,3,6-Trihydroxy-8-methylxanthone (**10**)

Yellowish needles (MeOH); M.p.: 228–230 °C; UV λ_max_ (MeOH) (log ε): 239 (4.24), 254 sh (3.96), 268 sh (3.68), 3.09 (4.00) nm; UV λ_max_ (MeOH + KOH) (log ε): 235 (4.23), 263 (4.03), 295 sh (3.59), 357 (4.40) nm; IR ν_max_ (ATR): 3511 (OH), 1655 (C=O) cm^−^^1^; ^1^H NMR (CD_3_OD, 400 MHz) δ 2.77 (3H, s, H-10), 6.11 (1H, d, *J* = 2.4 Hz, H-4), 6.22 (1H, d, *J* = 2.4 Hz, H-2), 6.59 (1H, d, *J* = 2.4 Hz, H-5), 6.60 (1H, d, *J* = 2.4 Hz, H-7); ^13^C NMR (CD_3_OD, 100 MHz) δ 23.6 (C-10), 94.2 (C-2), 98.8 (C-4), 101.5 (C-5), 104.0 (C-9a), 112.9 (C-8a), 117.0 (C-7), 144.7 (C-8), 158.6 (C-1), 160.8 (C-5a), 164.2 (C-6), 164.9 (C-4a), 166.1 (C-3), 183.5 (C-9); ESIMS *m*/*z* 259 [M + H]^+^.

#### 4.3.11. Ergosterol (**11**)

Colorless needles (CH_2_Cl_2_-MeOH); M.p.: 159–161 °C; IR ν_max_ (ATR): 3408 (OH) cm^−^^1^; ^1^H NMR (Acetone-*d*_6_, 600 MHz) δ 0.67 (3H, s, H-18), 0.85 (3H, d, *J* = 7.2 Hz, H-26), 0.86 (3H, d, *J* = 7.2 Hz, H-27), 0.94 (3H, d, *J* = 6.6 Hz, H-28), 0.95 (3H, s, H-19), 1.07 (3H, d, *J* = 6.6 Hz, H-21), 1.29 (1H, m, H-1b), 1.30 (1H, m, H-12b), 1.32 (1H, m, H-17), 1.38 (1H, m, H-16b), 1.39 (1H, m, H-15b), 1.46 (1H, m, H-2b), 1.49 (1H, m, H-25), 1.64 (1H, m, H-11b), 1.67 (1H, m, H-15a), 1.76 (1H, m, H-11a), 1.81 (1H, m, H-16a), 1.83 (1H, m, H-2a), 1.88 (2H, m, H-1a, H-24), 1.92 (1H, m, H-14), 1.96 (1H, m, H-9), 2.09 (2H, m, H-12a, H-20), 2.23 (1H, m, H-4b), 2.40 (1H, ddd, *J* = 14.4, 4.8, 2.4 Hz, H-4a), 3.50 (1H, m, H-3), 3.64 (1H, d, *J* = 4.8 Hz, OH-3), 5.24 (2H, dd, *J* = 15.6, 7.5 Hz, H-22), 5.28 (2H, dd, *J* = 15.6, 7.2 Hz, H-23), 5.37 (1H, m, H-7), 5.53 (1H, dd, *J* = 6.0, 3.0 Hz, H-6); ^13^C NMR (Acetone-*d*_6_, 150 MHz) δ 13.1 (C-18), 17.4 (C-19), 18.8 (C-28), 20.7 (C-26), 21.0 (C-27), 22.3 (C-21), 22.5 (C-11), 24.4 (C-15), 29.7 (C-16), 33.6 (C-2), 34.6 (C-25), 38.6 (C-10), 40.0 (C-1), 40.6 (C-12), 42.0 (C-20), 42.5 (C-4), 44.3 (C-13), 44.5 (C-24), 47.9 (C-9), 56.0 (C-14), 57.3 (C-17), 71.0 (C-3), 118.1 (C-7), 120.7 (C-6), 133.5 (C-23), 137.3 (C-22), 142.1 (C-5), 142.3 (C-8); ESIMS *m*/*z* 419 [M + Na]^+^.

#### 4.3.12. 6′-Hydroxy-4,2′,3′,4′′-tetramethoxy-*p*-terphenyl (**12**)

Colorless needles (MeOH); M.p.: 228–230 °C; UV λ_max_ (MeOH) (log ε): 209 (4.24), 227 sh (3.96), 274 (3.68) nm; UV λ_max_ (MeOH + KOH) (log ε): 212 (4.23), 233 sh (3.59), 276 (4.40) nm; IR ν_max_ (ATR): 3446 (OH) cm^−^^1^; ^1^H NMR (CDCl_3_, 400 MHz) δ 3.45 (3H, s, H-3′), 3.75 (3H, s, H-2′), 3.85 (3H, s, H-4′′), 3.88 (3H, s, H-4), 5.93 (1H, br s, OH-3′, D_2_O exchangeable), 6.47 (1H, s, H-5′), 7.00 (2H, d, *J* = 8.4 Hz, H-3′′, 5′′), 7.41 (2H, d, *J* = 8.8 Hz, H-2, 6), 7.59 (2H, d, *J* = 8.8 Hz, H-2′′, 6′′); ESIMS *m*/*z* 367 [M + H]^+^.

#### 4.3.13. Palmitic Acid (**13**)

Whitish powder; IR ν_max_ (ATR): 3300–2700 (OH), 1706 (C=O) cm^−^^1^; ^1^H NMR (CDCl_3_, 400 MHz) δ 0.88 (3H, t, *J* = 6.8 Hz, H-16), 1.25 (24H, s, H-4–15), 1.63 (2H, quint, *J* = 7.4 Hz, H-3), 2.34 (2H, t, *J* = 7.4 Hz, H-2); EIMS *m*/*z* 256 [M]^+^ (44), 227 (12), 213 (35)_,_ 199 (21), 185 (35), 171 (36),157 (42), 143 (28), 129 (67), 115 (31), 87 (100), 73 (80), 57 (52).

### 4.4. Determination of NO Cell Viability Assay

#### 4.4.1. NO Activity Assay

The murine macrophage cell line RAW 264.7 cells obtained from the Bioresource Collection and Research Center (BCRC; Hsinchu, Taiwan), were cultured in Dulbecco’s modified Eagle medium (DMEM) supplemented with 10% fetal calf serum (FCS) and penicillin–streptomycin, and incubated at 37 °C in a humidified atmosphere with 5% CO_2_.

The nitrite measurement was based on our published technique [26]. A cell density of 2 × 10^5^ cells/100 μL were seeded into 96-well plates for 24 h. After pre-treating the cells with the compounds, a positive control, aminoguanidine (a selective iNOS inhibitor; 100 μM), or the DMSO (0.1%) vehicle before 200 ng/mL of lipopolysaccharide (LPS) induction. Twenty-four hours after LPS induction, the culture supernatant was collected and assayed for nitrite concentration by the Griess reagent. The absorbance at 550 nm was measured with a microplate spectrophotometer (Bio-Tek Instrument, Inc., Winooski, VT, USA). Fresh medium was used as the blank. The results were expressed as a percentage of the LPS induction without compound treatment group.

#### 4.4.2. Cell Viability Assay

After the culture supernatant had been removed for nitrite determination, we used the MTT (3-(4,5-dimethylthiazol-2-yl)-2,5-diphenyltetrazolium bromide) solution to assess the cell viability as previously described [27]. In brief, 0.5 mg/mL of MTT in DMEM was added to each well of cells and incubated in cell culture condition for 3 h. Then, the supernatant was removed from each well by suction, and 100 μL of DMSO was added to dissolve the formazan crystals. The absorbance at 570 nm was measured with a microplate spectrophotometer. The results were expressed as a percentage of the LPS induction without compound treatment group.

## Figures and Tables

**Table 1:** ^1^H (400 MHz) and ^13^C (100 MHz) NMR data of **1**, **2**, and **5** (CDCl_3_); ^1^H (600 MHz) and ^13^C (150 MHz) NMR data of **3** (CDCl_3_); δ in ppm, *J* in Hz.

Position	1		2		3		5	
	δ (*J* in Hz)	δ_C_	δ (*J* in Hz)	δ_C_	δ (*J* in Hz)	δ_C_	δ (*J* in Hz)	δ_C_
2		167.2		167.7		165.0		168.2
3		115.1		115.6		116.8		115.1
4		178.4		178.3		178.2		179.2
4a		116.5		115.9		116.5		115.6
5	8.03 d (8.8)	124.1	6.90 s	101.8	8.03 d (8.9)	124.4	7.90 d (8.8)	123.8
6	6.92 d (8.8)	108.0		156.2	6.94 d (8.9)	108.2		114.2
7		160.6	7.96 s	127.1		160.8	7.03 d (8.8)	159.5
8		113.3		123.4		113.2		111.4
8a		156.0		159.4		154.8		155.9
9	3.00 m	37.6	3.00 m	37.5	3.11 dq (7.2, 6.6)	44.9	3.05 m	37.8
10	1.82 m	27.7	1.77 m	27.5	4.15 dq (6.6, 6.0)	70.2	1.85 m	27.8
	1.68 m		1.64 m				1.68 m	
11	0.89 t (7.4)	11.9	0.89 t (7.6)	12.0	1.34 d (6.0)	21.1	0.93 dd (7.6, 7.2)	12.0
12	1.29 d (6.8)	18.0	1.27 d (6.8)	17.8	1.32 d (7.2)	14.6	1.33 d (6.8)	18.0
13	2.05 s	9.4	2.09 s	9.7	2.09 s	9.7	2.11 s	9.6
14	2.27 s	7.8	2.33 s	15.6	2.29 s	8.0	2.35 s	7.9
OH-6			7.35 br s					
OH-7							8.91 br s	
OCH_3_-7	3.91 s	55.9			3.94 s	56.0		

**Table 2:** Inhibitory effects of the seven isolates from *L*. *abnorme* on LPS-activated NO production in RAW 264.7 macrophages ^a^.

Compounds	Nitrite Production (%)	*E*_max_ (%) ^b^	Cell Viability (%)
lachnochromonin F (**3**)	88.48 ± 6.45	11.52 ± 6.45	98.17 ± 13.65
lachabnormic acid (**4**)	82.46 ± 1.67	17.54 ± 1.67	73.62 ± 7.68
alternariol-9-methyl ether (**7**)	101.83 ± 0.69	−1.83 ± 0.69	83.00 ± 4.67
alternariol (**8**)	86.65 ± 2.22	13.35 ± 2.22	106.41 ± 8.19
pestalorionol (**9**)	91.62 ± 2.24	8.38 ± 2.24	107.33 ± 5.87
1,3,6-trihydroxy-8-methyl-xanthone (**10**)	83.77 ± 0.53	16.23 ± 0.53	96.40 ± 11.07
palmitic acid (**13**)	77.75 ± 2.89	22.25 ± 2.89	92.83 ± 0.13
aminoguanidine	35.59 ± 0.2	78.74 ± 0.64	88.87 ± 2.98

^a^ Results of these are shown as the mean ± standard error of means (SE) from three independent experiments; ^b^ *E*_max_ indicates mean maximum inhibitory effect of nitrite production at a concentration of 100 μM. iNOS inhibitor aminoguanidine is a positive control.

## Supplementary Materials

Supplementary materials can be found at www.mdpi.com/1422-0067/17/9/1512/s1.
