# Supplementary material for: Secondary Metabolites of the Endophytic Fungus Lachnum abnorme from Ardisia cornudentata
Source: Int J Mol Sci. 2016 Sep 8;17(9):1512. doi: 10.3390/ijms17091512 (PMC5037789; doi:10.3390/ijms17091512)
Supplement: Supplementary file 1 [file ijms-17-01512-s001.pdf]

Supplementary Materials: Secondary Metabolites of the Endophytic Fungus *Lachnum abnorme* from *Ardisia cornudentata*

Hsun-Shuo Chang, Chu-Hung Lin, Yi-Shuan Chen, Hui-Chun Wang, Hing-Yuen Chan, Sung-Yuan Hsieh, Ho-Cheng Wu, Ming-Jen Cheng, Gwo-Fang Yuan, Shan-Yu Lin, Yue-Jin Lin and Ih-Sheng Chen

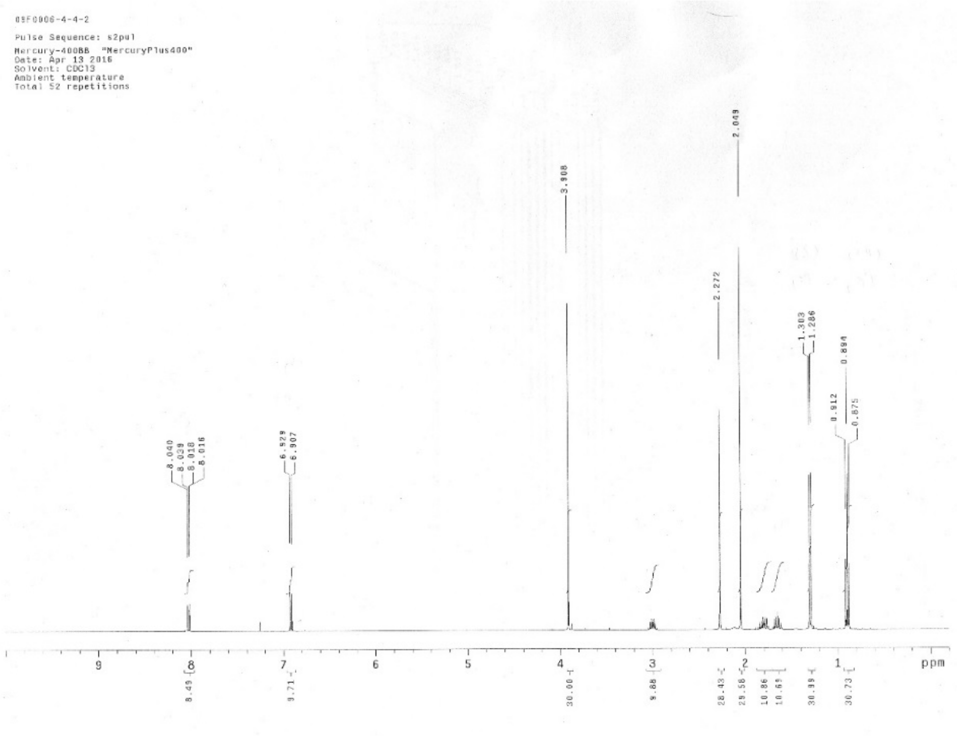

Figure S1. ^{1}H NMR spectrum of lachnochromonin C (**1**) in $CDCl_3$ at 400 MHz.

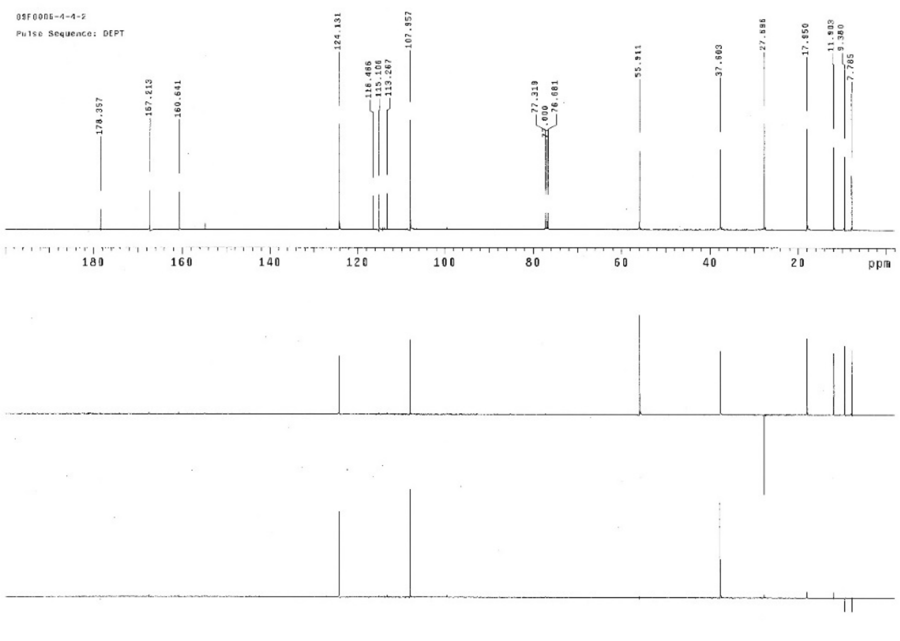

Figure S2. DEPT spectrum of lachnochromonin C (**1**) in $CDCl_3$ at 100 MHz.

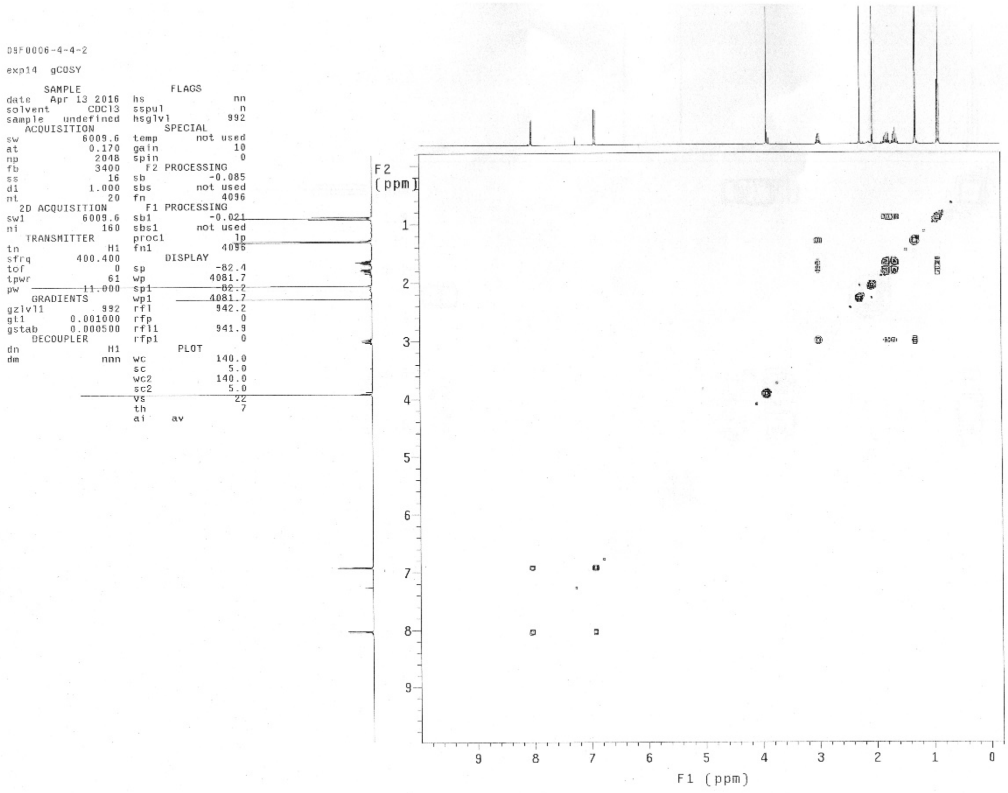

Figure S3. COSY spectrum of lachnochromonin C (**1**).

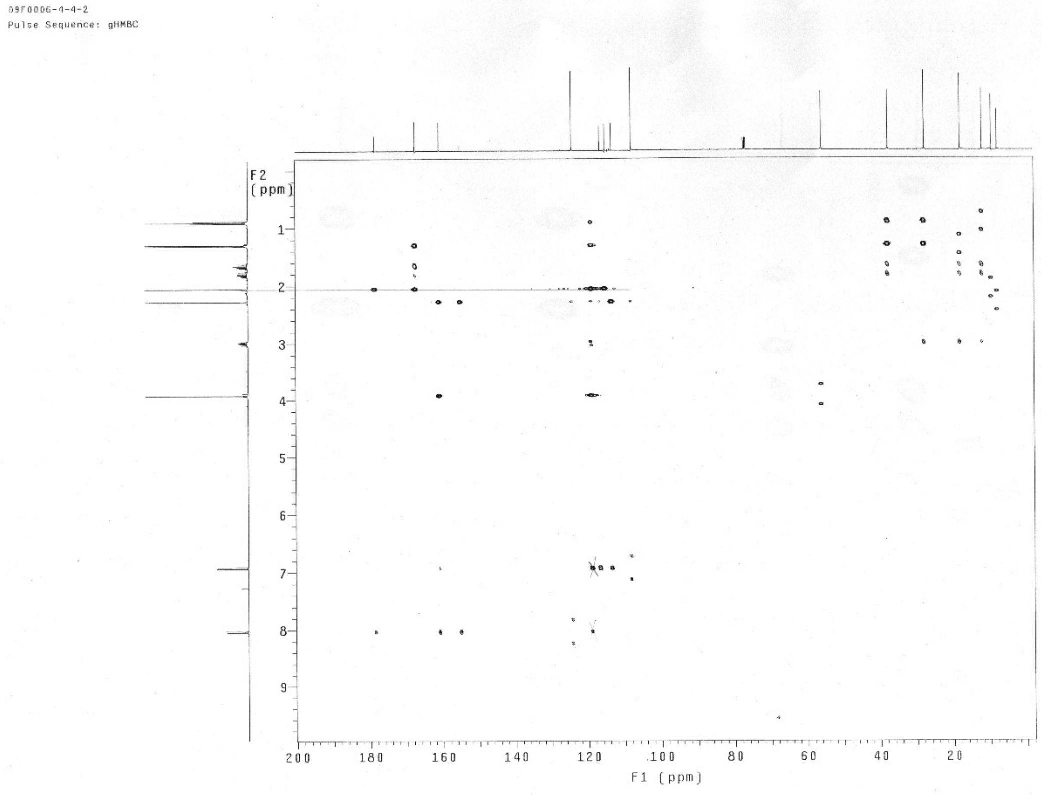

Figure S4. HMBC spectrum of lachnochromonin C (**1**).

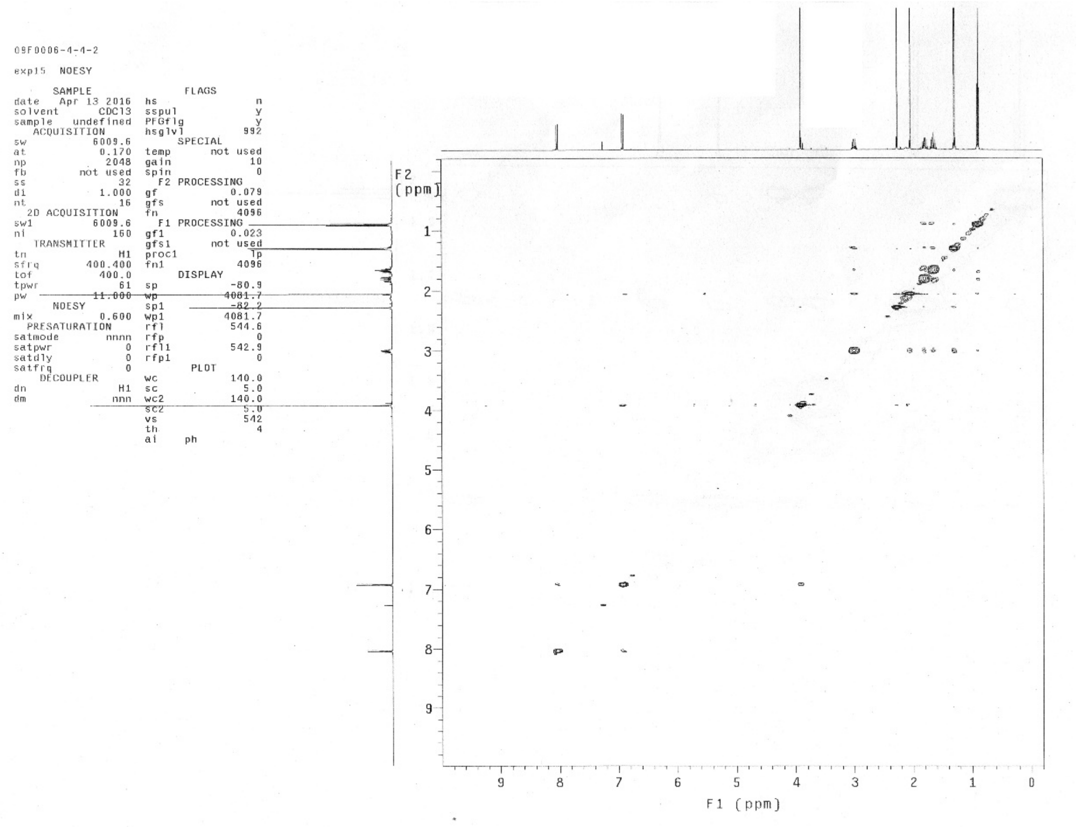

Figure S5. NOESY spectrum of lachnochromonin C (**1**).

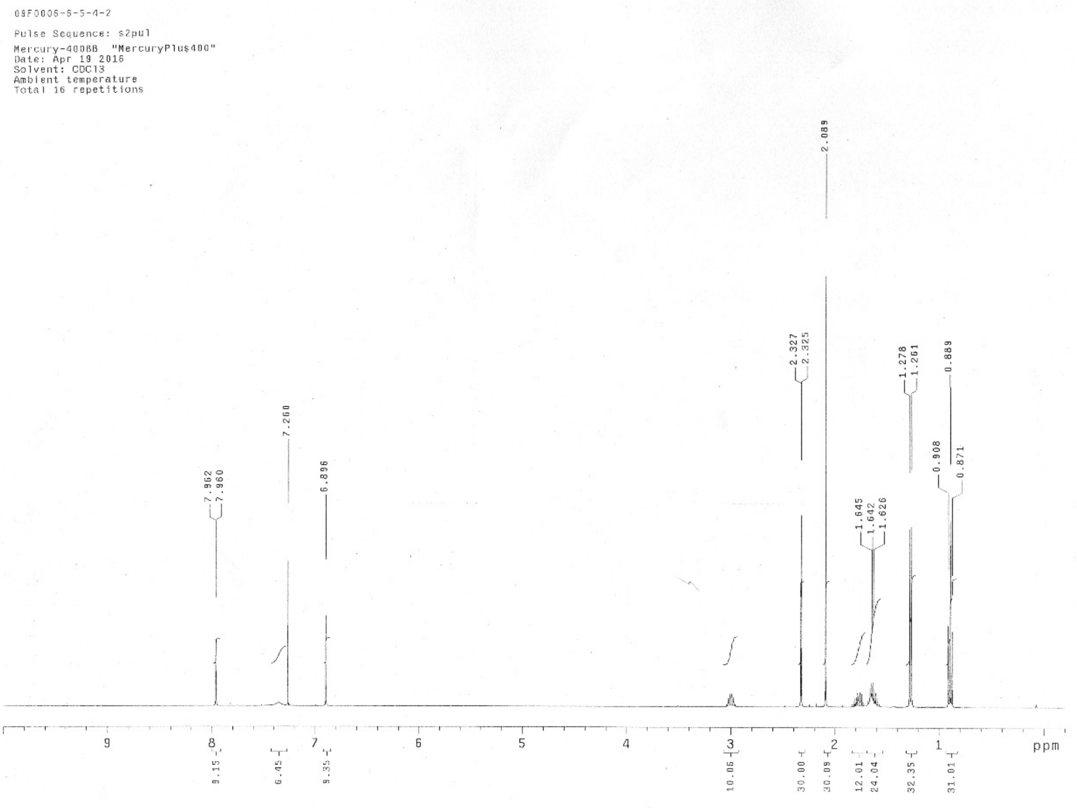

Figure S6. ^{1}H NMR spectrum of lachnochromonin D (**2**) in $CDCl_3$ at 400 MHz.

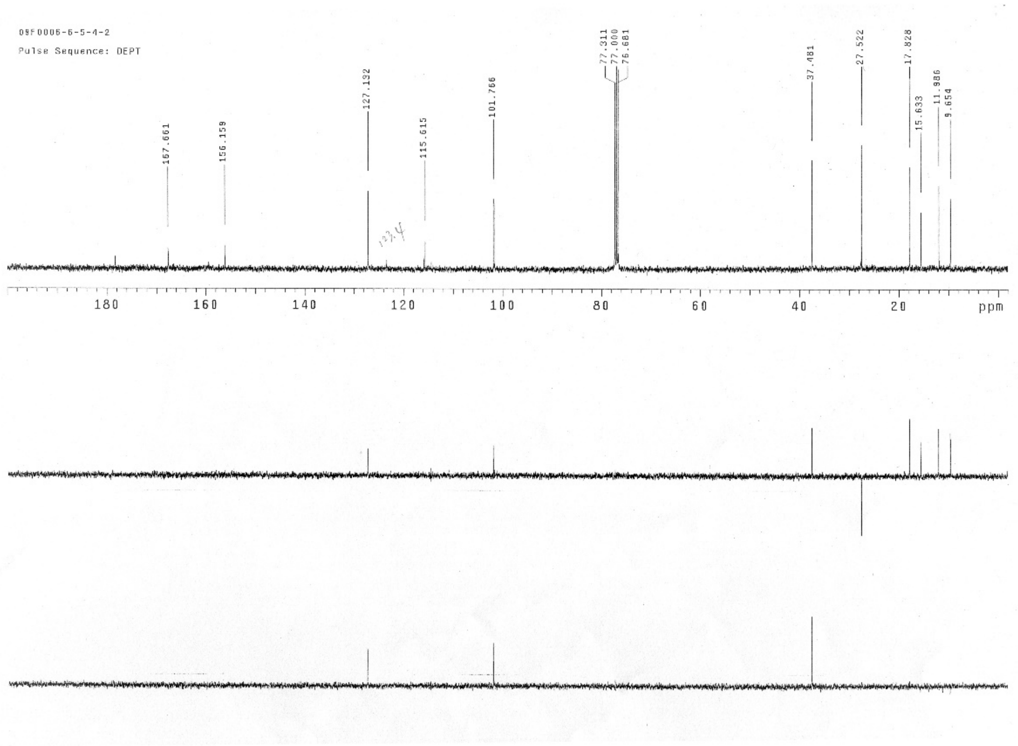

Figure S7. DEPT spectrum of lachnochromonin D (**2**) in $CDCl_3$ at 100 MHz.

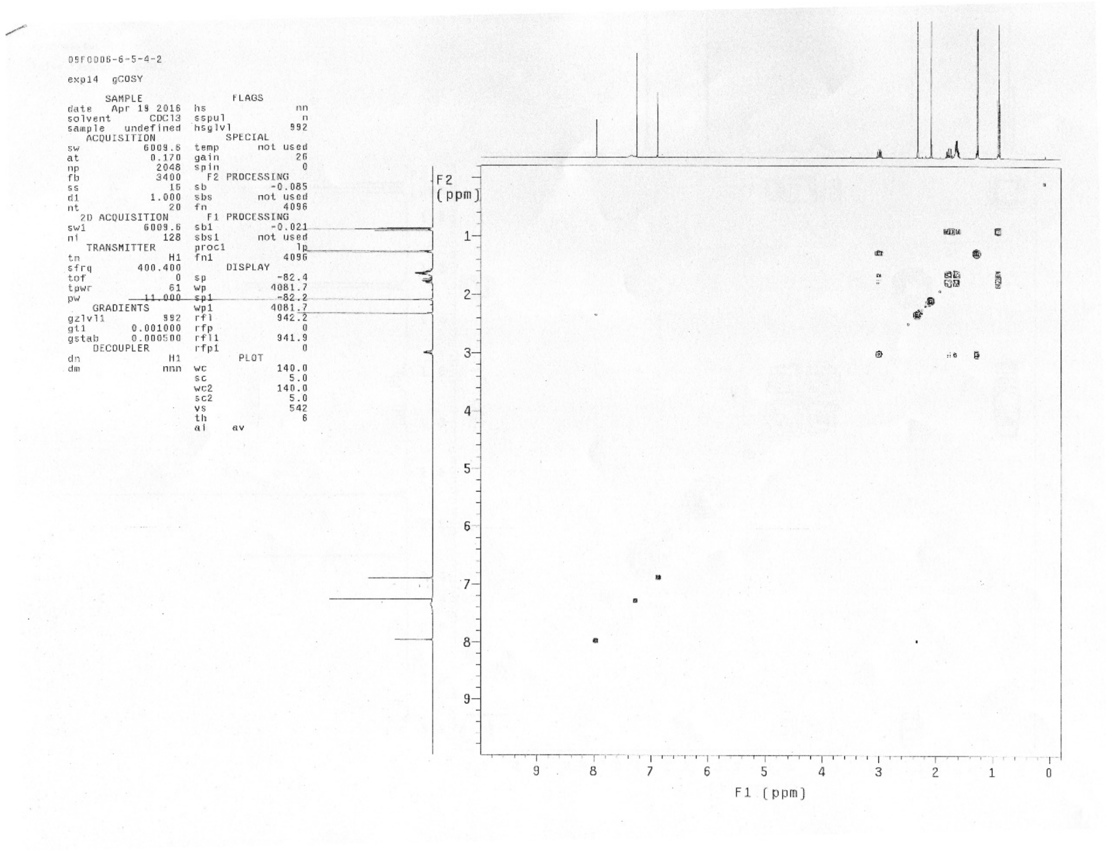

Figure S8. COSY spectrum of lachnochromonin D (**2**).

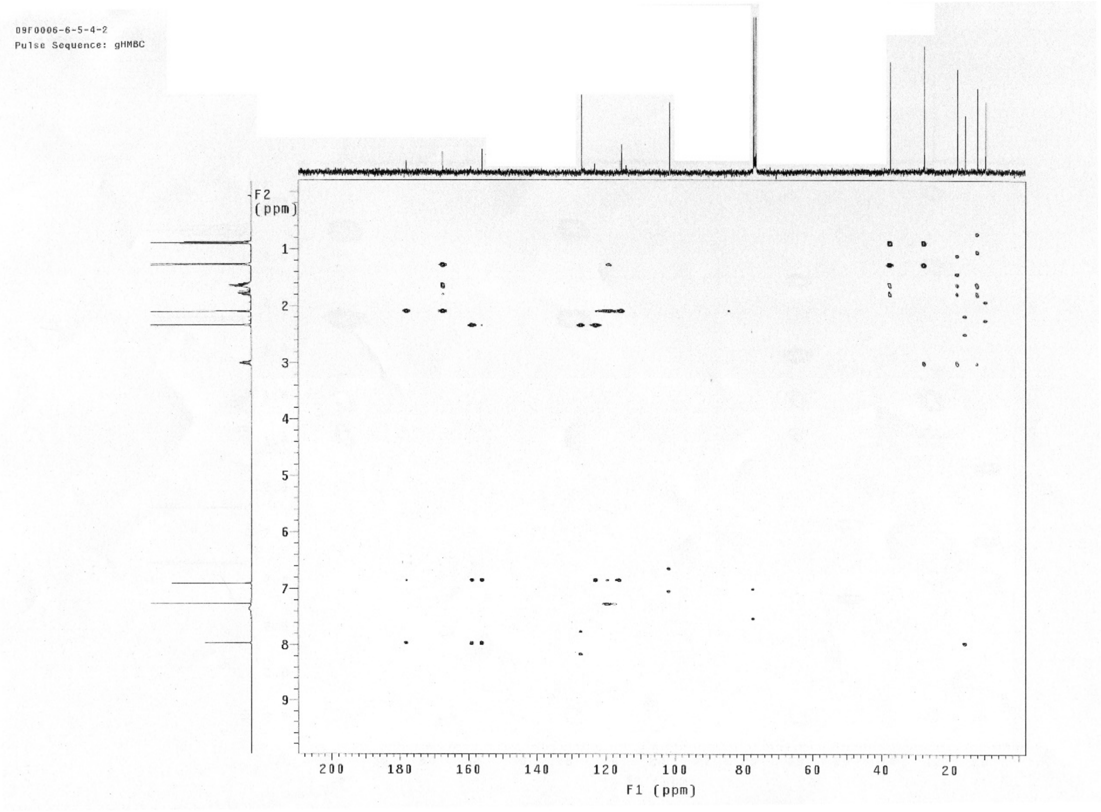

Figure S9. HMBC spectrum of lachnochromonin D (**2**).

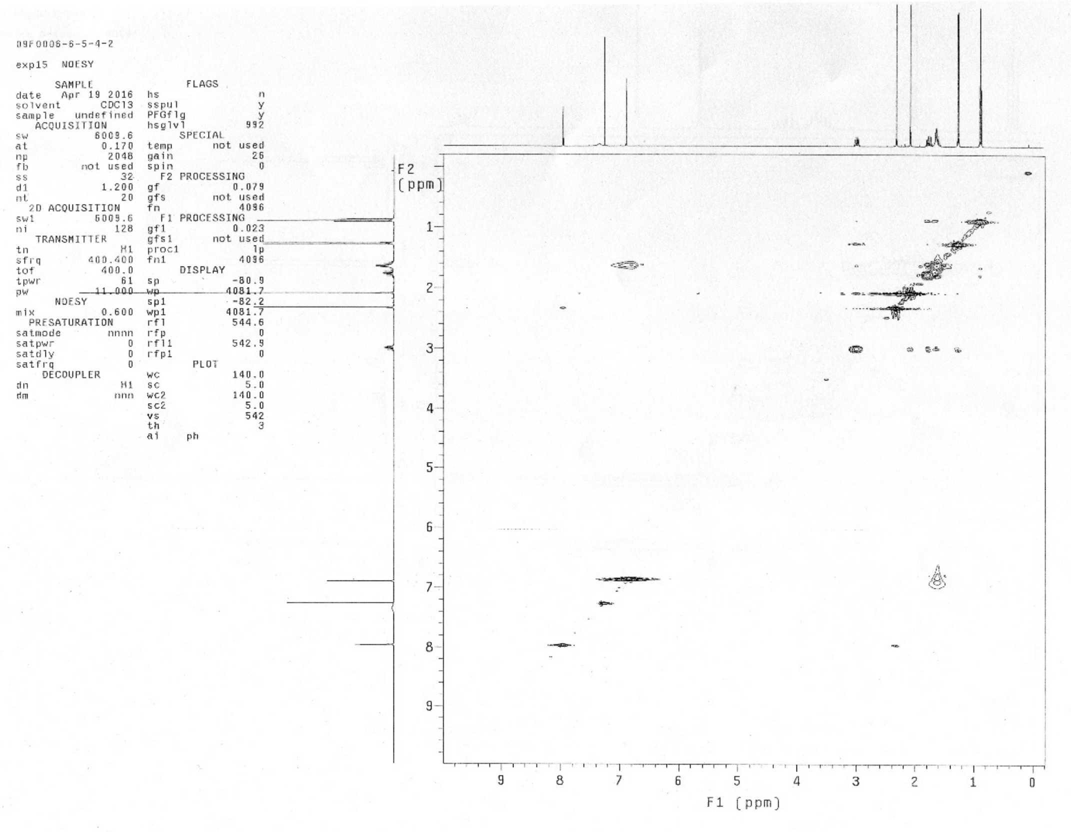

Figure S10. NOESY spectrum of lachnochromonin D (**2**).

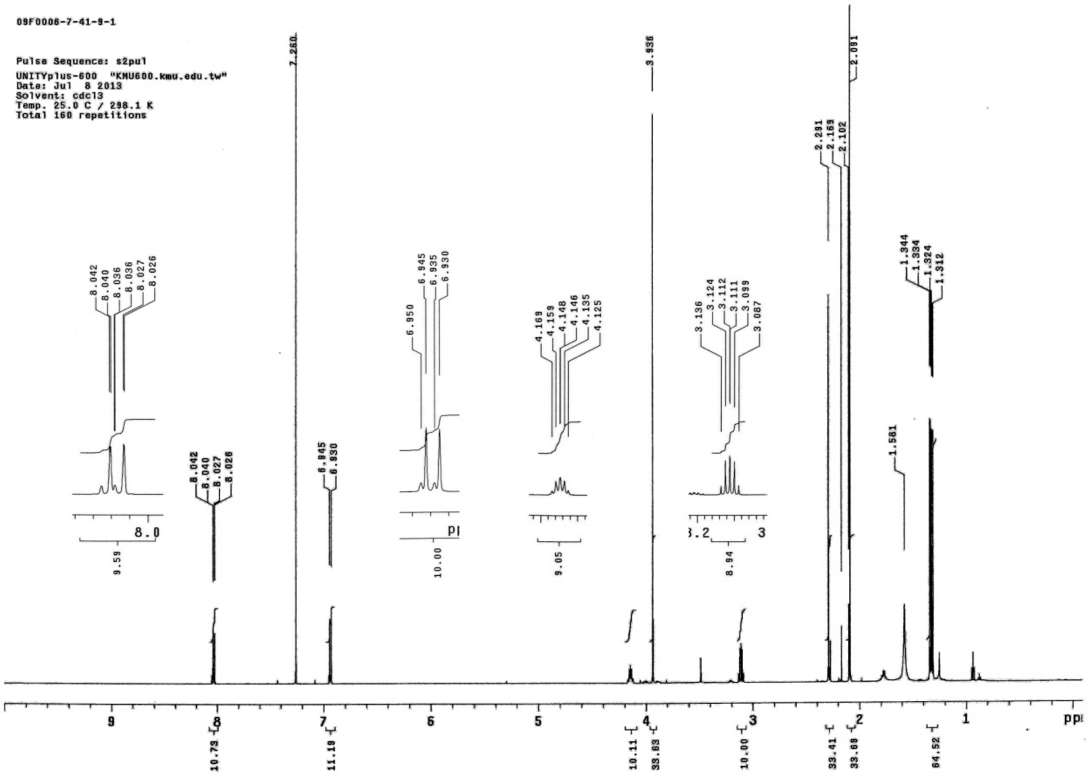

Figure S11. ^{1}H NMR spectrum of lachnochromonin E (**3**) in $CDCl_3$ at 600 MHz.

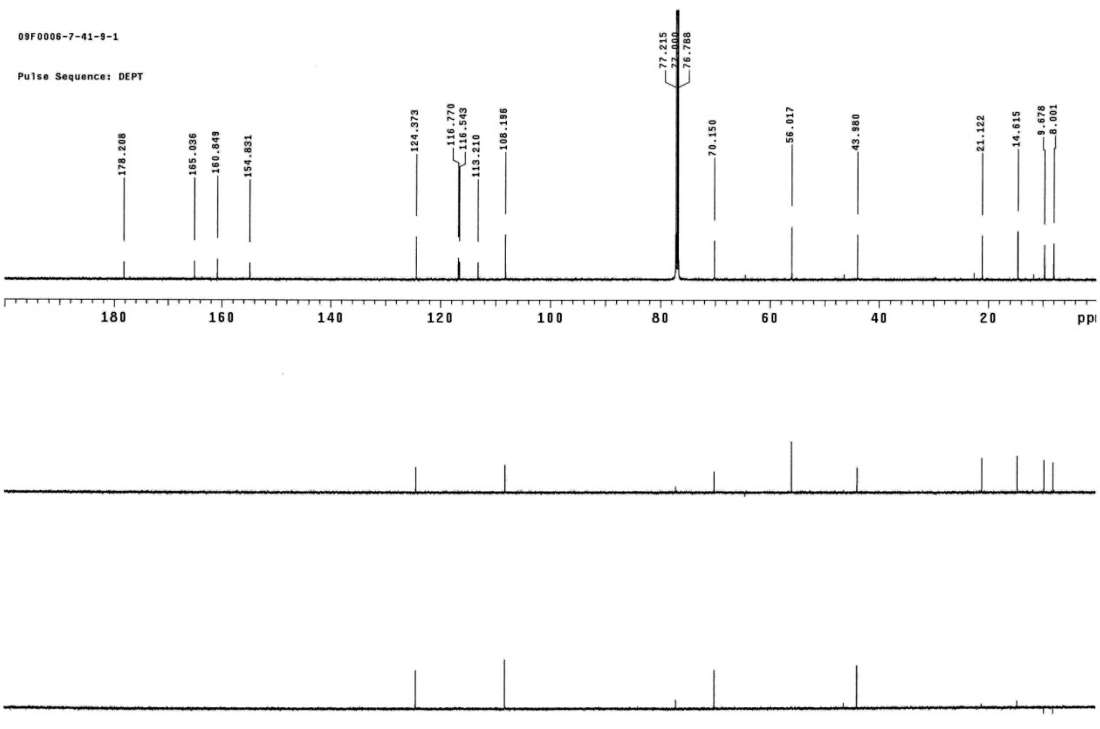

Figure S12. DEPT spectrum of lachnochromonin E (**3**) in $CDCl_3$ at 150 MHz.

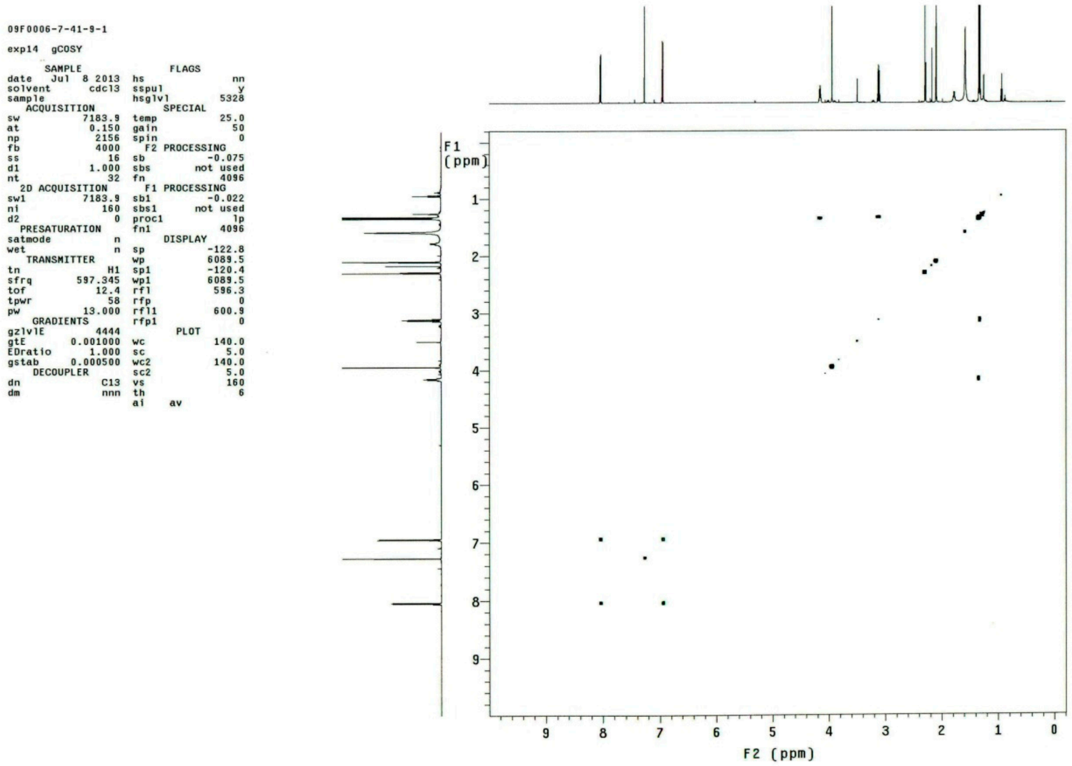

Figure S13. COSY spectrum of lachnochromonin E (**3**).

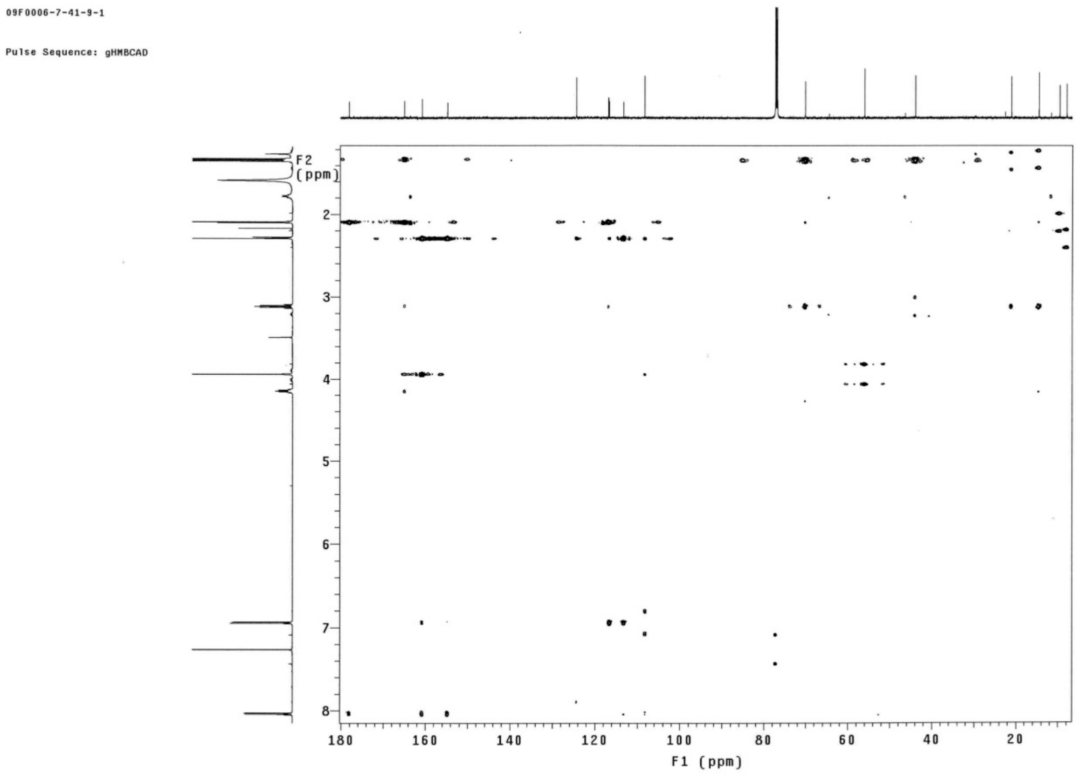

Figure S14. HMBC spectrum of lachnochromonin E (**3**).

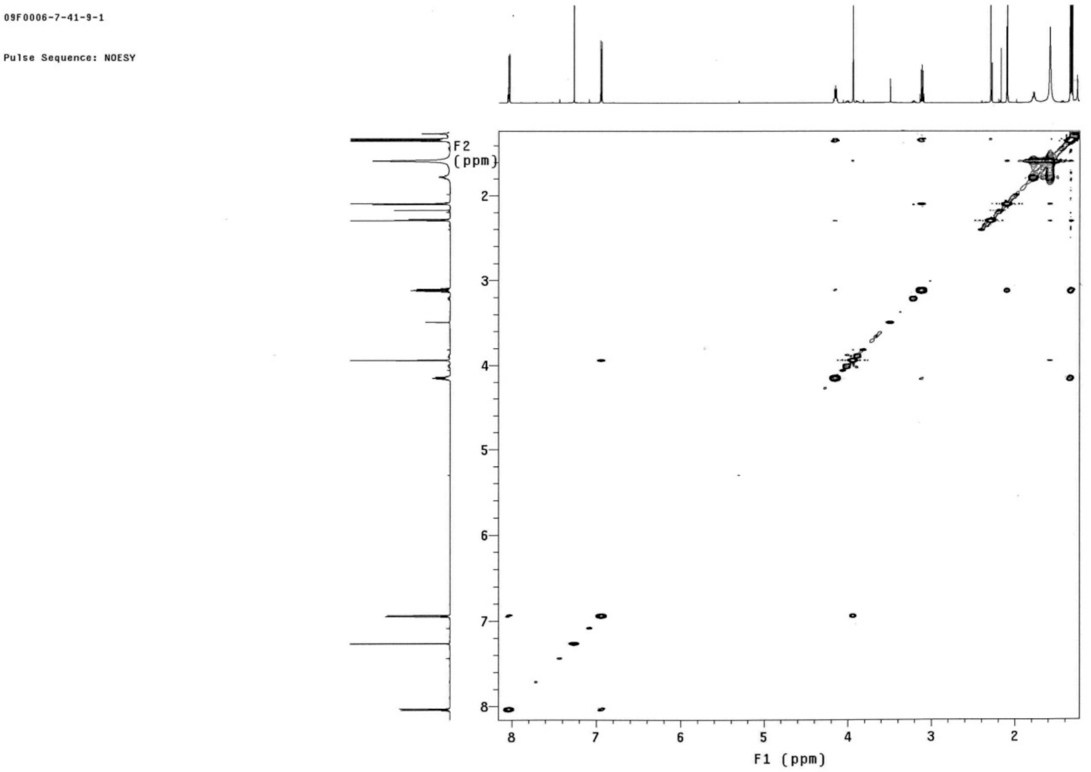

Figure S15. NOESY spectrum of lachnochromonin E (**3**).

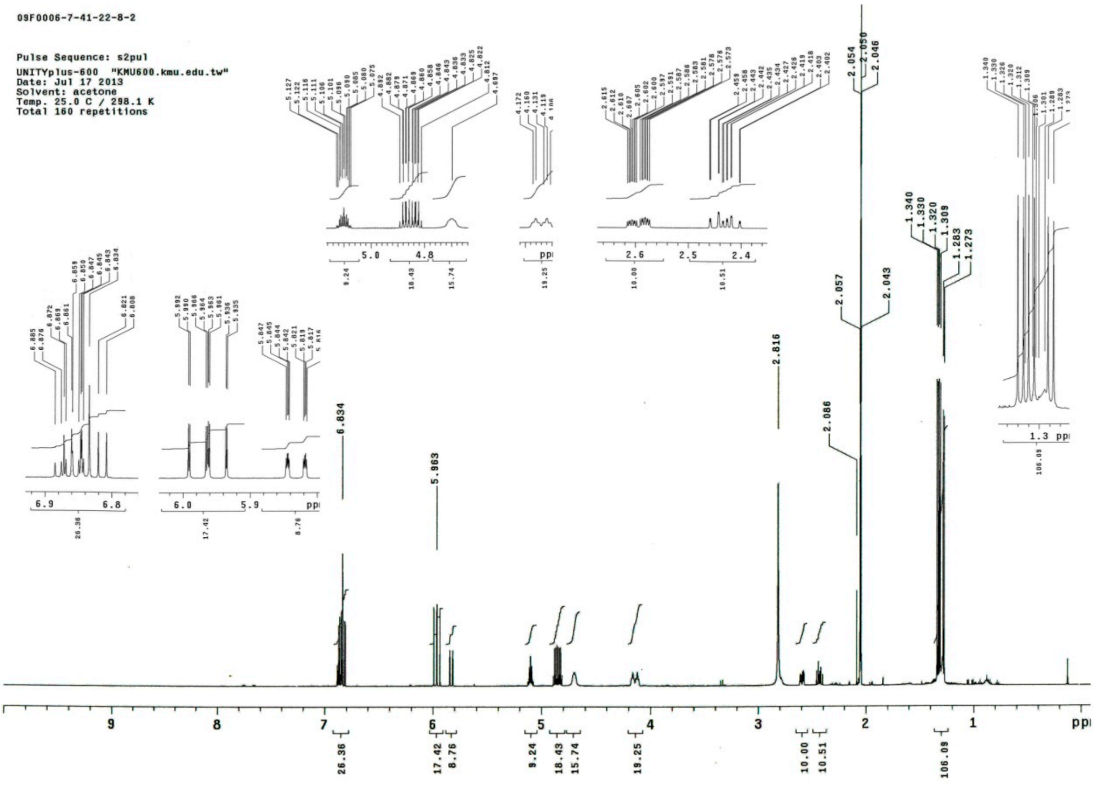

Figure S16. ^{1}H NMR spectrum of lachabnormic acid (**4**) in acetone-d_6 at 600 MHz.

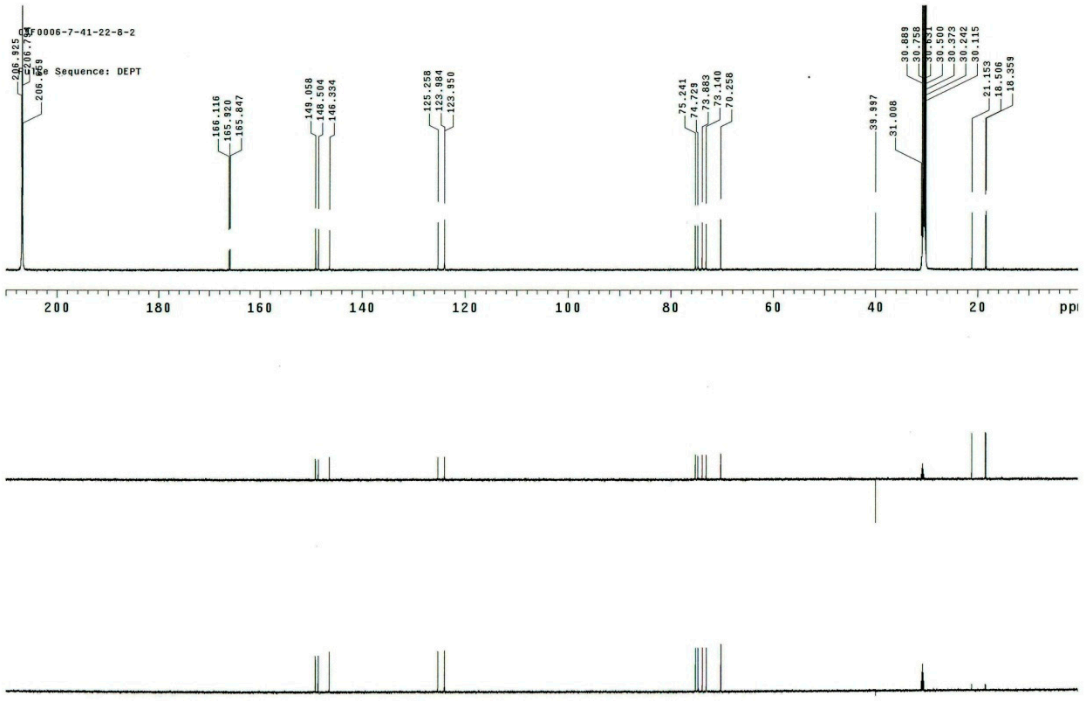

Figure S17. DEPT spectrum of lachabnormic acid (**4**) in acetone-d_6 at 150 MHz.

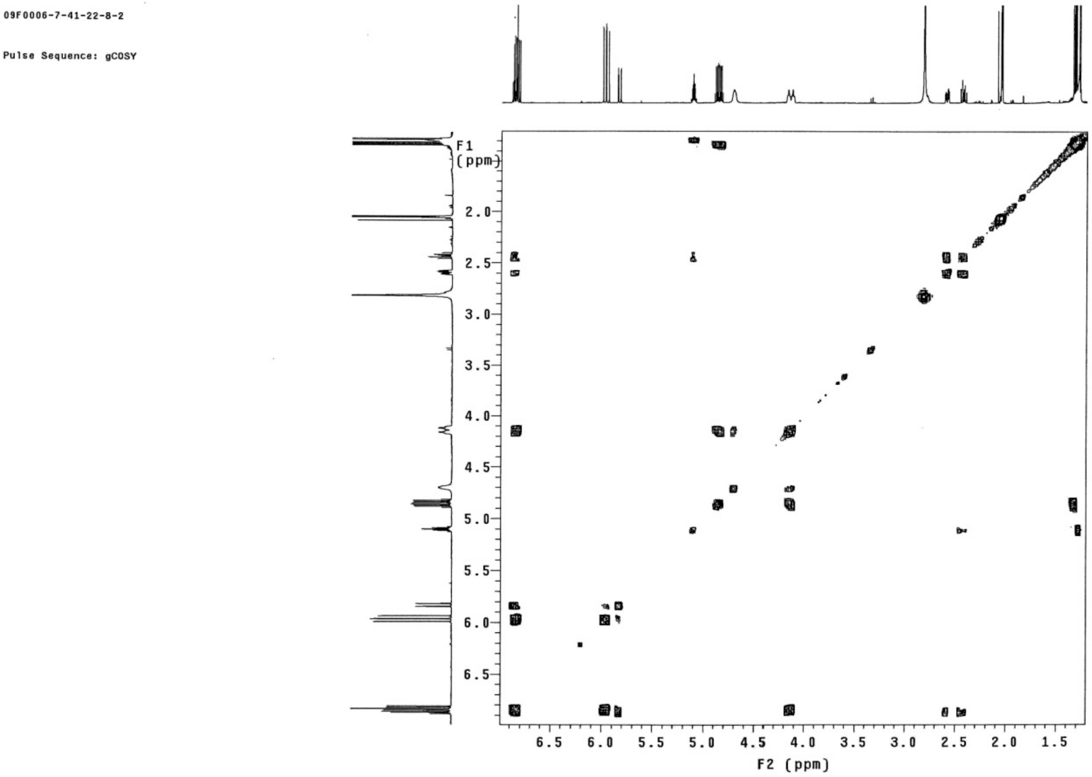

Figure S18. COSY spectrum of lachabnormic acid (**4**).

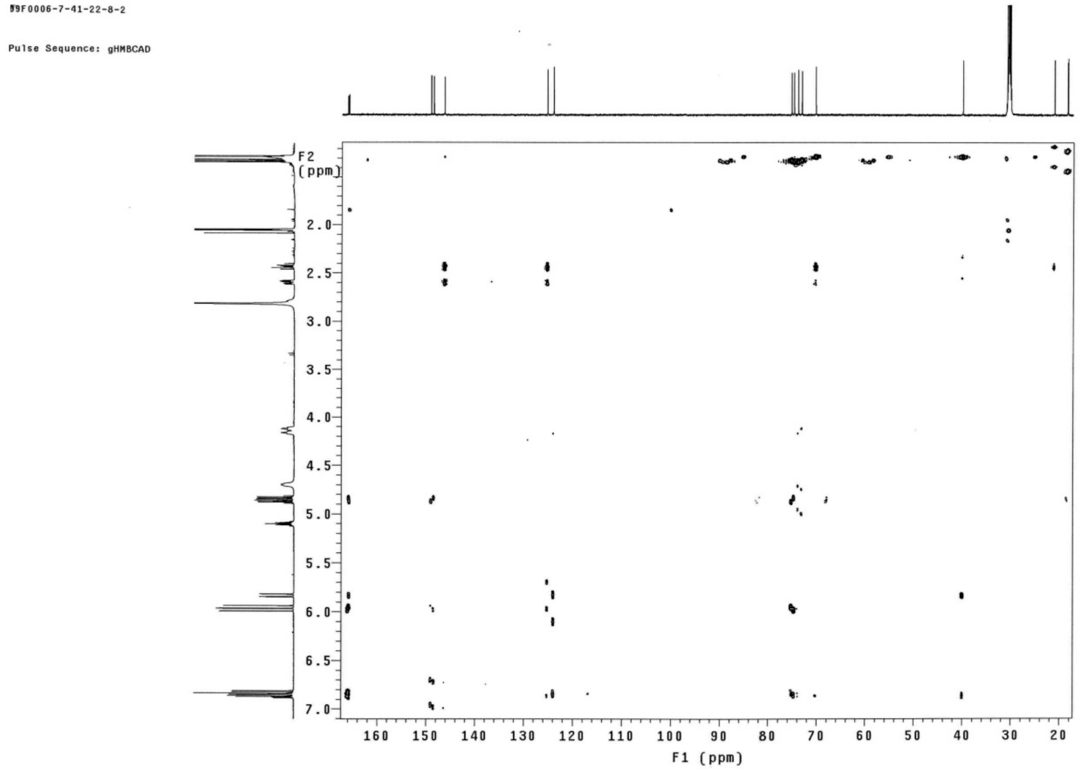

Figure S19. HMBC spectrum of lachabnormic acid (**4**).

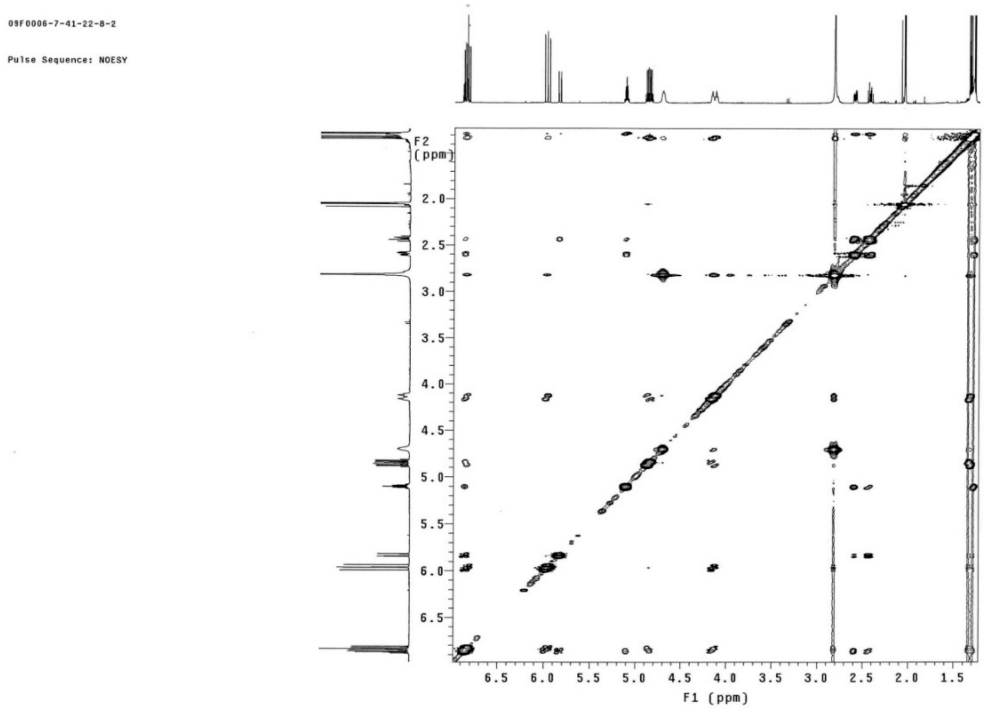

Figure S20. NOESY spectrum of lachabnormic acid (**4**).

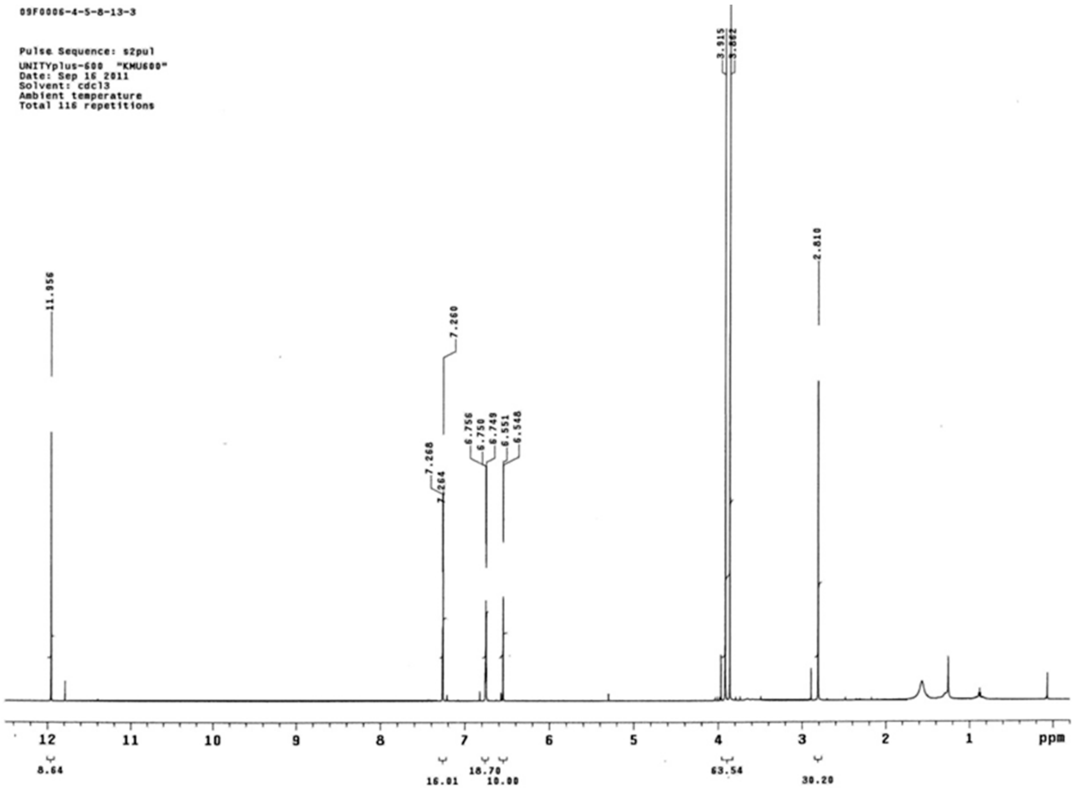

Figure S21. ^{1}H NMR spectrum of alternariol-3,9-dimethyl ether (**6**) in $CDCl_3$ at 600 MHz.

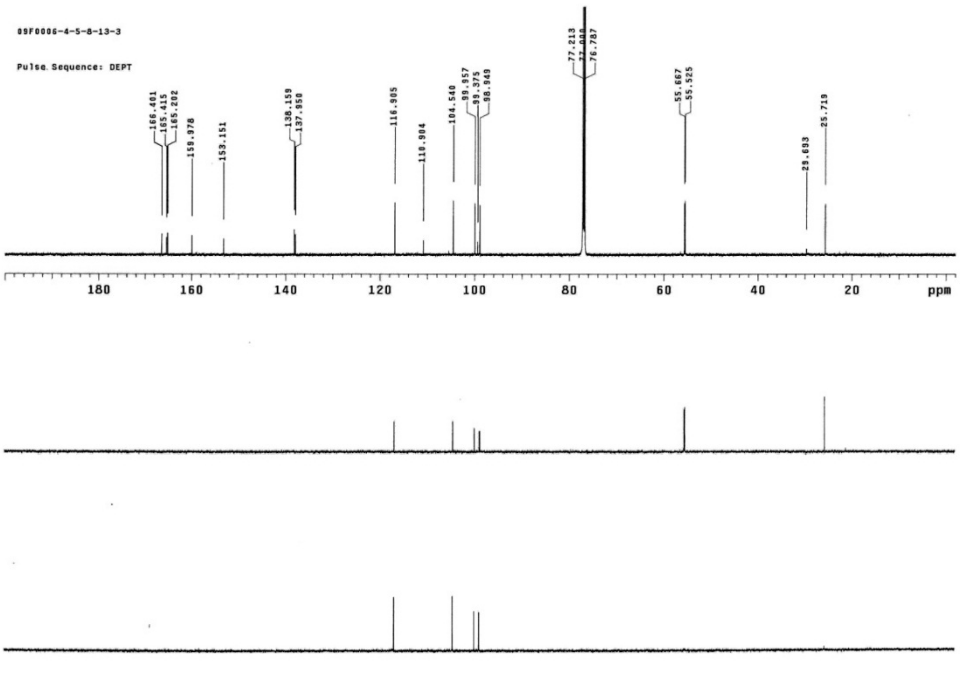

Figure S22. DEPT spectrum of alternariol-3,9-dimethyl ether (**6**) in $CDCl_3$ at 150 MHz.

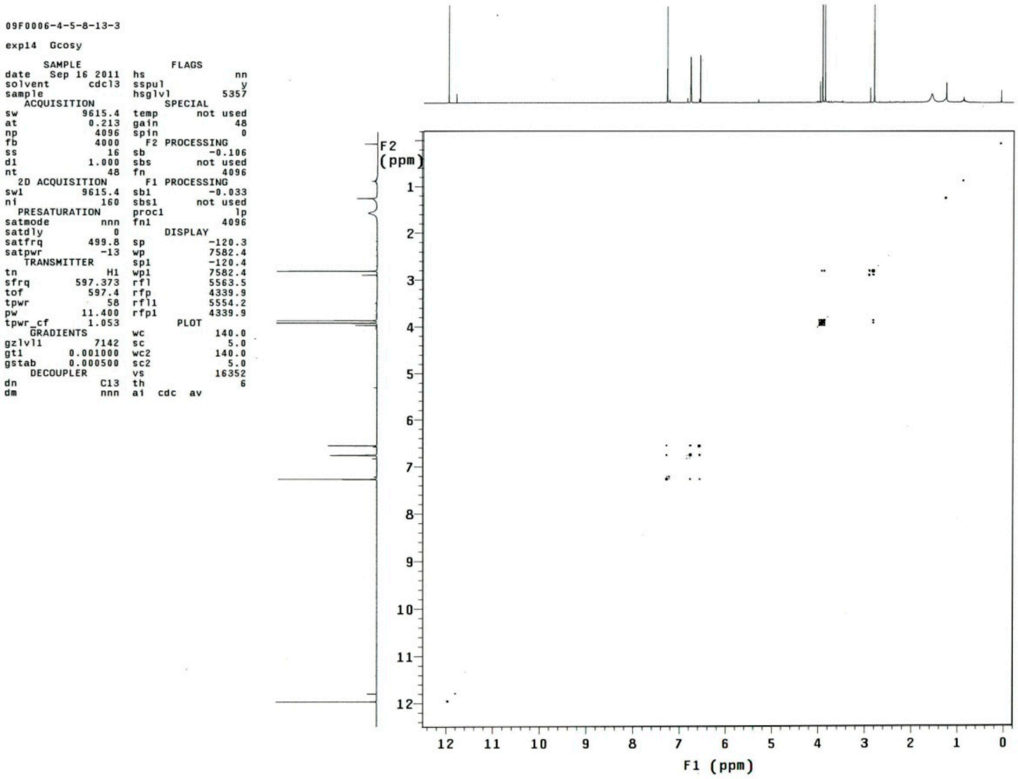

Figure S23. COSY spectrum of alternariol-3,9-dimethyl ether (**6**).

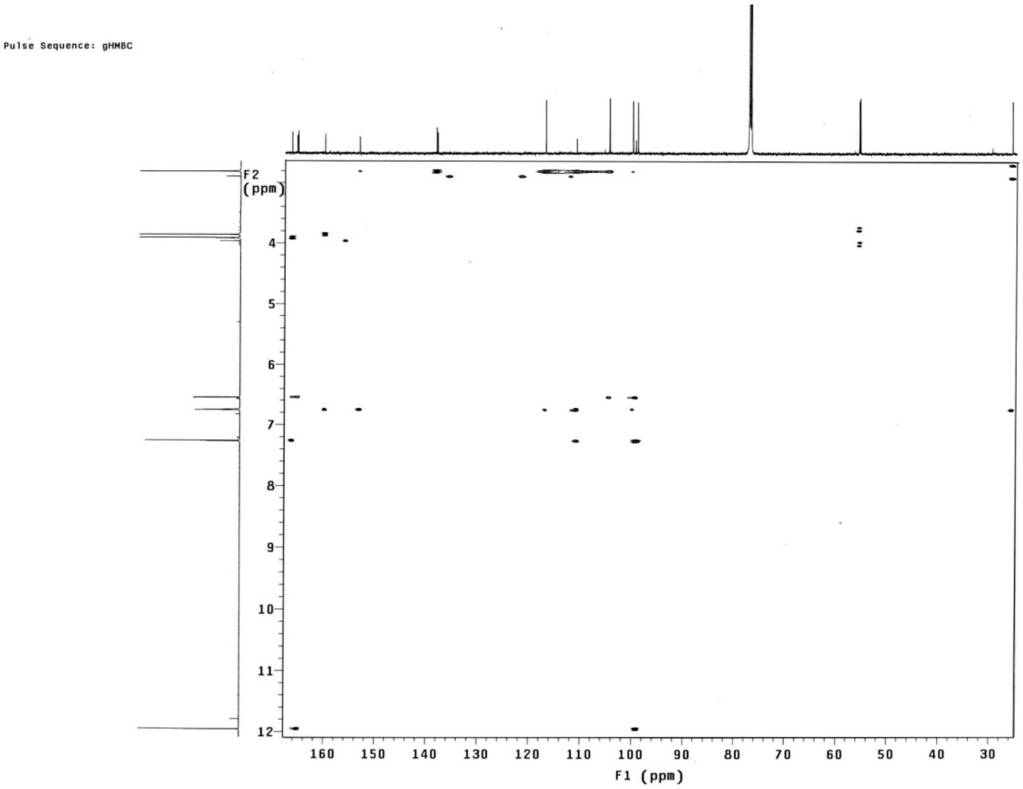

Figure S24. HMBS spectrum of alternariol-3,9-dimethyl ether (**6**).

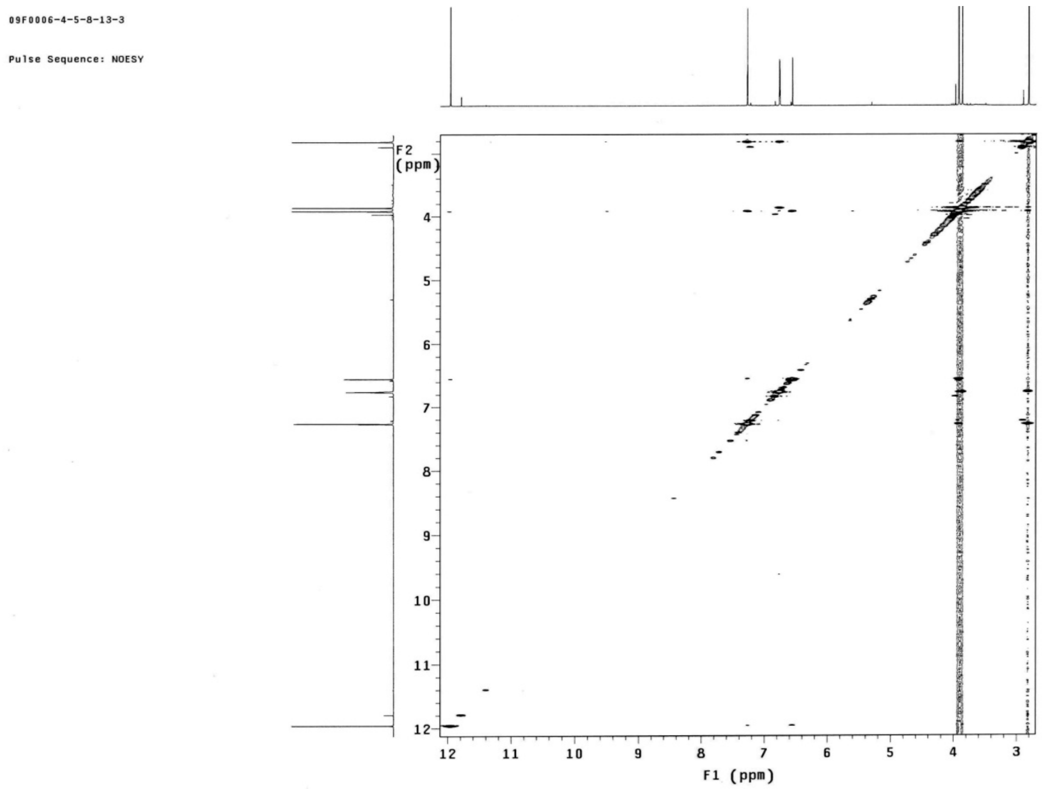

Figure S25. NOESY spectrum of alternariol-3,9-dimethyl ether (**6**).

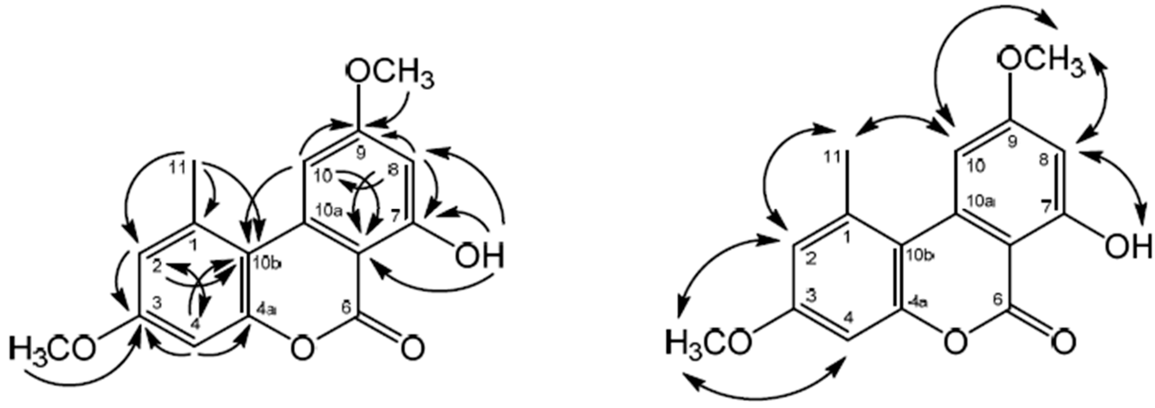

Figure S26. Key HMBC and NOESY correlations for compound **6**.
